# Compressing Few-Cycle Optical Near Fields in the Tip–Sample Junction of a Scanning Probe Microscope

**DOI:** 10.1021/acs.nanolett.5c06074

**Published:** 2026-02-11

**Authors:** Tom Jehle, Sam S. Nochowitz, Juanmei Duan, Christoph Lienau

**Affiliations:** Institut für Physik, 11233 Carl von Ossietzky Universität, 26129 Oldenburg, Germany

**Keywords:** Ultrafast scattering-type near-field spectroscopy, ultrafast near-field dynamics, few-cycle optical fields, spectral interferometry, plasmonic nanogaps

## Abstract

Plasmonic nanogaps confine light to dimensions in the nanometer or even subnanometer range while simultaneously enhancing the local electromagnetic field strength. This spatial light confinement has been key for the development of nano-optics. So far, the temporal dynamics of such nanoconfined fields has received comparatively little attention, in particular in the visible spectral range. Here we measure the amplitude and phase of the electric field of visible to near-infrared light pulses scattered from the gap between a sharp gold tip and a metal surface. We retrieve the time structure of the field with subcycle precision. We provide evidence for a complex-valued local near-field enhancement and demonstrate that the spatial confinement of a few-cycle pulse in the investigated nanogap is correlated with a substantial reduction in its pulse duration. Our results pave the way for probing the linear and nonlinear electric field dynamics of single quantum emitters in nanogaps.

## Graphical abstract

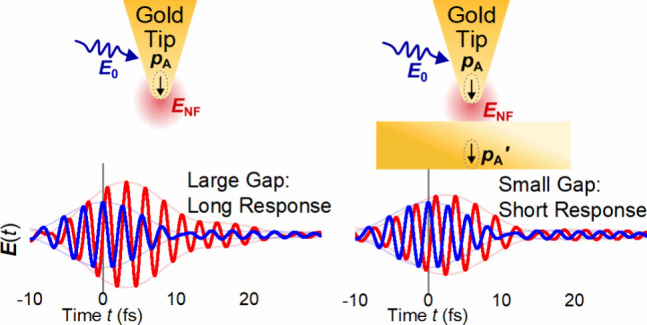

The spatial confinement of light to a nanoscale volume in the gap between a plasmonic nanostructure and a surface and the resulting field enhancement have been instrumental for the development of nano-optics during the past four decades,^1^ leading to significant advances in our understanding of light–matter interactions on the nanoscale.^2–4^ A variety of nanogap geometries, ranging from the tip–sample junction of a scanning probe microscope^5,6^ over nanoparticles on mirrors (NPoM),^3,7–9^ to kissing metal spheres^4,10^ have been introduced, reaching light localization on the angstrom scale and field enhancements of up to 100–1000. Such nanogaps have found numerous applications over the past decade, including surface-enhanced Raman spectroscopy (SERS),^8,11^ (bio)­sensing,^12,13^ and studies of the coupling of single molecules to gap modes^3,14–16^ and of the effects of electron tunneling and nonlocal screening^4,10,17^ on the light scattering spectra from subnanometer gaps. Freestanding, sharp conical metallic tapers reach a somewhat lower field enhancement in the range of 10.^18,19^ Such tapers offer flexible control over the nanogap formation, instrumental for the development of a wide range of scanning probe microscopy (SPM) techniques like scanning tunneling microscopy (STM)-induced luminescence,^5,20,21^ ultrafast STM,^22–25^ tip-enhanced Raman spectroscopy,^2,26–29^ and scattering-type scanning near-field optical microscopy (s-SNOM).^30–33^ In addition, coherent electron microscopy techniques such as photon-induced near-field electron microscopy^34^ are rapidly developing as high-spatial-resolution local probes of optical near-fields.^35–37^

Whereas the spatial confinement of nanogap fields has received much attention in the research community, experimental studies of the temporal dynamics of spatially highly localized fields are still scarce. Such measurements, although experimentally challenging, would be highly desirable, for example, for exploring the coherent coupling of quantum emitters to gap modes,^3,15,16,38^ for advancing SPM-based ultrafast coherent phonon spectroscopy,^39,40^ and for nonlinear nanoscale optics.^41^ Recent work in this direction successfully recorded the dynamics of few-cycle terahertz fields emitted from a few-angstrom tip–sample junction,^23^ and extensions of such studies to the visible range are currently underway.^25,42^ So far, however, amplitude- and phase-resolved spectroscopic studies of broadband light scattering from tip–sample junctions are mostly restricted to the infrared spectral range. They use Fourier transform spectroscopy^43–46^ or pseudoheterodyne SNOM^47,48^ as phase-sensitive detection methods yet, so far, do not retrieve full information about the local electric field dynamics.

Here we use high-repetition-rate spectral interferometry to measure the amplitude and phase of few-cycle visible to near-infrared light pulses that are scattered from the junction between a gold surface and a sharp gold tip. By varying the tip–sample distance we observe near-field-mediated spectral red shifts and line broadenings which allow us to quantify the local field enhancement. We retrieve the time structure of the local field at the tip apex with subcycle precision and show that the coupling of the tip to its image dipole results in a substantial reduction in pulse duration when the field is confined in the gap.

Experimentally, we introduce a broadband spectral interferometry technique to probe the amplitude and phase of the light that is scattered from the tip–sample junction of an s-SNOM (Figure 1a). For this, we focus few-cycle pulses from a Ti:sapphire laser with an 80 MHz repetition rate onto the apex of a sharp gold taper. By using an all-reflective microscope objective with a numerical aperture of 0.4, the pulse duration of 9 fs (full width at half-maximum of the intensity profile) is maintained in the ∼1 μm-sized focal region, as confirmed by a separate frequency-resolved interferometric autocorrelation measurement using an α-BBO crystal instead of the tip (see Supporting Information (SI) section 3).^49,50^ The light field that is scattered from the tip, the signal field **E**_S_(*t*), is recorded in back reflection through the same objective and focused into a single-mode fiber (SMF). Here, it is overlaid with a reference field **E**_R_(*t*) = *E*_R_(*t*)**ê**_*z*_ = *t*_R_(*t*) ⊗ **E**_0_(*t* – τ_0_) that is linearly polarized along **ê**_*z*_. **E**_R_ is a replica of the incident field **E**_0_(*t*) time-delayed by τ_0_ and slightly distorted by its transmission through the interferometer with transmission coefficient *t*_R_(*t*). The Kronecker product (⊗) denotes the convolution of the two quantities. Both the signal and reference field are passed through a monochromator and a spectral interferogram (SI), given by^51,52^

1 $$S\left(\omega \right)=I_{R}\left(\omega \right)+I_{S}\left(\omega \right)+E_{R}\left(\omega \right)E_{S}^{*}\left(\omega \right)e^{i\omega \tau_{0}}+E_{R}^{*}\left(\omega \right)E_{S}\left(\omega \right)e^{-i\omega \tau_{0}}$$

is recorded using a fast line scan camera. The intensity spectra of the reference field and signal field are *I*_R_(ω) = |**E**_R_(ω)|^2^ and *I*_S_(ω) = |**E**_S_(ω)|^2^, respectively. The detected signal field amplitude is *E*_S_(ω) = **E**_S_(ω)·**ê**_*z*_. The repetition rate of the camera (E2V Aviiva EM4), 219 kHz, is sufficiently high to record phase-stable interferograms, unaffected by mechanical instabilities of the setup. The camera sensitivity of ∼60 photons/count allows us to probe the weak picowatt-level fields^32^ that are scattered out of the gap junction.

**Figure 1:**
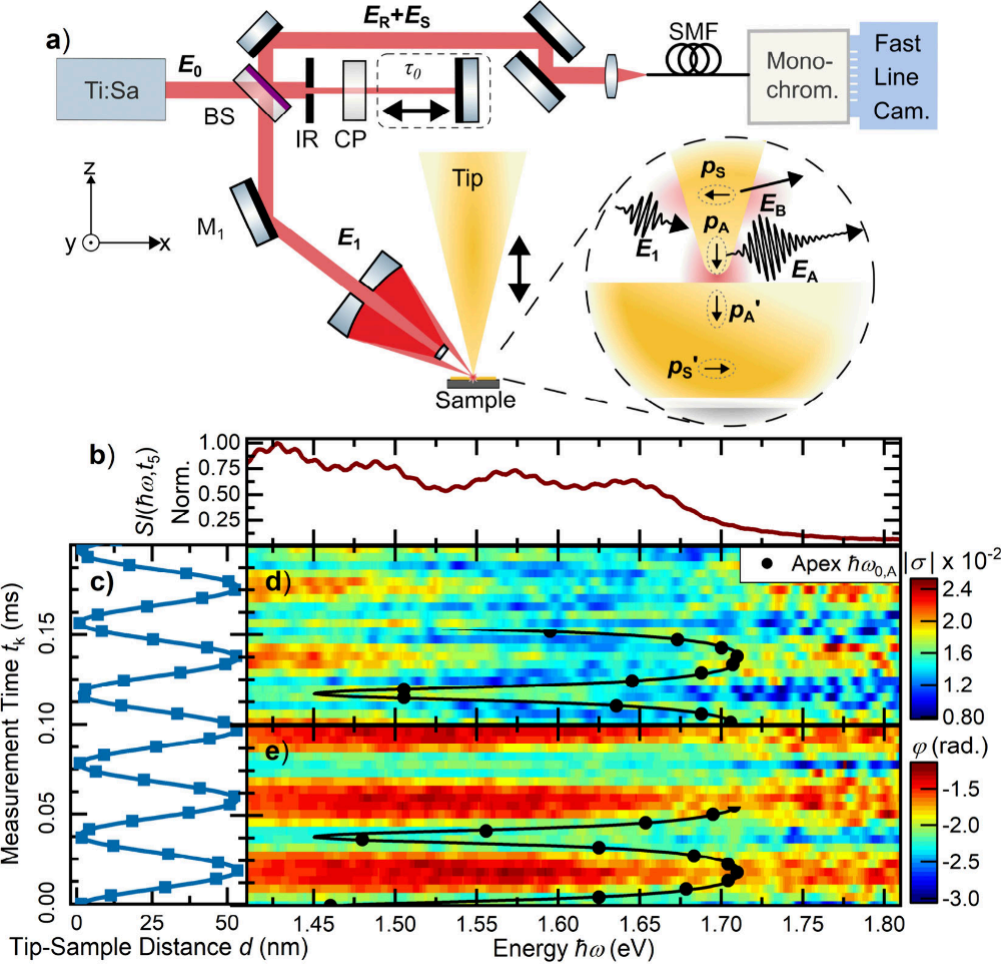
(a) Schematic setup of spectral interferometry scanning near-field microscopy (SI-SNOM). The p-polarized light from a 9 fs Ti:sapphire laser is focused onto the junction between the gold taper and gold surface with periodic modulation of the tip–sample distance. Light that is backscattered from the junction, **E**_S_, is collected, overlapped with the reference field **E**_R_ in a single-mode fiber (SMF), spectrally dispersed in a monochromator, and imaged onto a fast line camera to generate a spectral interferogram (SI). The interferometer contains a beamsplitter (BS), an iris (IR), and a dispersion compensation plate (CP). The inset schematically depicts the relevant dipole moments induced at the taper apex, **p**_A_, and the shaft, **p**_S_, together with their mirror images (**p**_A_^′^, **p**_S_^′^) and their emitted far fields **E**_A_ and **E**_B_. (b) Typical SI with 10% fringe contrast, recorded at *d*(*t*_5_) = 53 nm. (c) Time-dependent tip–sample distance modulation *d*(*t*_*k*_). (d, e) Corresponding amplitude |σ­(ω)| and spectral phase φ­(ω) of the response function σ­(ω) retrieved from the SIs. The data are overlaid in the bottom halves with the distance-dependent resonance energy of the apex mode, ℏω_0,A_ (black line), as a guide to the eye, indicating the strong red shift during the approach of the tip to the gold surface.

We start by analyzing light scattering from the gap between a gold taper and a planar gold film with 250 nm thickness. The gold tip is formed by electrochemical etching^53^ and has an apex radius of *a*_0_ = 10 nm and a half-opening angle of θ = 18° (see SI section 2). We illuminate the tip–sample junction with linearly p-polarized light and record light scattering spectra as a function of tip–sample distance *d*, periodically modulating the distance at a tapping frequency of *f*_t_ ≈ 25 kHz with a peak-to-peak amplitude of 52 nm. By measuring the tapping force, we set the minimum distance to approximately 1 nm.

While modulating the tip–sample distance, we continuously record SIs of the scattered light at the full repetition rate of the camera, i.e., one SI per 4.6 μs. In these measurements, the delay τ_0_ is set to −340 fs. A characteristic SI is depicted in Figure 1b, showing the spectrum of the reference laser in the range from 1.41 to 1.81 eV,^54^ spectrally modulated by the interference with the weak signal field. The average modulation period of ∼12.5 meV is inversely proportional to τ_0_. The modulation contrast, taken as the difference between the signal intensities for constructive and destructive interference, is approximately 10% of the reference intensity across the entire spectral range. The measurements are performed with an average power of the detected reference field of ∼3 μW, while the total scattered field amounts to ∼2 nW. We note that such a high modulation contrast is reached because the SMF acts a spatial mode filter, suppressing light scattering from outside the apex region. In a single measurement, we record 30,000 consecutive SIs during a total measurement time of 0.15 s.

For each of these spectra, we extract the complex-valued spectral response function σ­(ω) = |σ­(ω)|e^i^ ^φ^ ^(ω)^ = *E*_S_(ω)/*E*_0_(ω) that connects the amplitude and phase of the signal and the incident field. For this, we use a Fourier domain analysis^54,55,57^ that is described in SI section 5. Time-dependent variations of amplitude and spectral phase are shown in Figure 1d,e, respectively. The data are compared to simultaneously recorded measurements of the tip–sample distance, shown as blue squares in Figure 1c. The blue line depicts the expected periodic tip–sample distance modulation.

Whenever the tip is far away from the gold surface, we find doubly peaked spectra with maxima near the lower- and higher-energy ends of the spectral range. When approaching the surface, the high-energy peak shows a red shift from 1.70 to 1.45 eV, correlated with a line broadening. The black circles denote the resonance energy of this peak as deduced from a Lorentzian oscillator model introduced below. The solid black lines depict the periodic variation of the resonance energy with sample distance. Also, the spectral phase φ­(ω) shows distinct periodic modulation with tapping frequency. These distance-dependent line shape changes become more clearly visible after data averaging over a few thousand modulation cycles, as shown in Figure 2.

**Figure 2:**
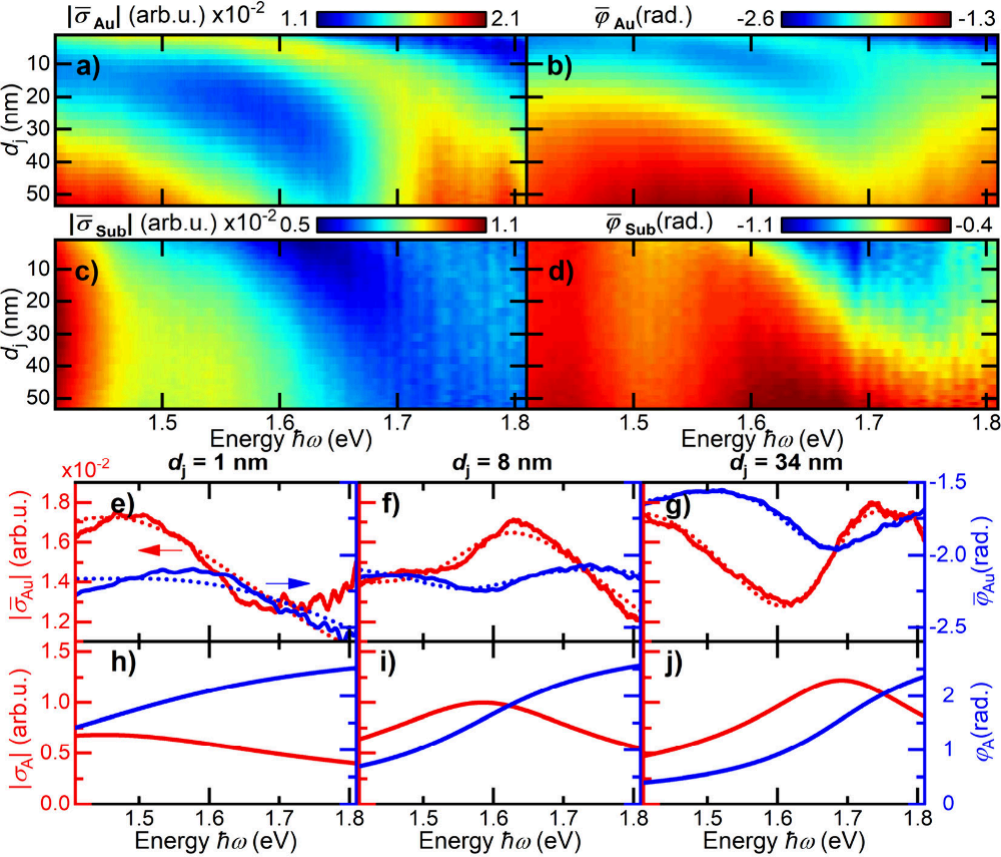
Spectral response σ̅(*d*, ℏω) recorded as a function of tip–sample distance *d* as the tip approaches a planar gold surface (Au) and a 90 nm dielectric SiO_2_ layer on a Si substrate (Sub). (a) Amplitude |σ̅_Au_| (a) and (b) spectral phase φ̅_Au_ recorded when approaching the gold surface. (c) Amplitude |σ̅_Sub_| and (d) spectral phase φ̅_Sub_ measured on the dielectric substrate. The light that is scattered from the apex region shows a pronounced red shift of both amplitude and phase of σ̅ as the tip approaches the gold surface. This spectral shift is essentially absent on the dielectric substrate. (e–g) Spectral dependence of |σ̅_Au_| (red) and φ̅_Au_ (blue) at selected distances *d*_*j*_ of 1, 8, and 34 nm. Fits to a Lorentz oscillator model are shown as red and blue dotted lines, respectively. (h–j) Amplitude σ̅_A_ and phase arg­(σ_A_) of the apex mode as extracted from the Lorentz oscillator model, shown as red and blue lines, respectively. The apex mode spectrally broadens when the tip approaches the surface.

For such data analysis, a tip–sample distance *d* is assigned to each of the scattering spectra by using the curve in Figure 1c. Then the spectra are sorted as a function of *d* and grouped into 32 bins of 1024 spectra each. An average over all spectra in one bin gives the distance-dependent spectral response σ̅(ω, *d*_*j*_) as a function of the tip–sample distance *d*_*j*_ of bin *j*. The amplitude and spectral phase of σ̅(ω, *d*_*j*_) are shown in Figure 2a,b, respectively. Most noticeably, the spectral amplitude shows a strong scattering resonance that is centered, for large distances, around 1.75 eV. Upon approach to the sample, this resonance red-shifts by more than 200 meV, in particular during the last 10 nm, i.e., when increasing the near-field coupling between tip and the gold substrate. This red shift is correlated with a change in the phase profile in the energy region of the resonance in Figure 2b. In addition, the response function reveals a second resonance in the red range of the spectrum. Its amplitude decreases gradually upon approach, while its resonance energy appears to be only weakly affected by near-field coupling.

When the gold layer is replaced with a purely dielectric substrate (Figure 2c,d), a silicon substrate covered with a 90 nm thick silicon dioxide layer, scattering resonances appear again in the same spectral range. Now, the blue resonance, resolved in both amplitude and spectral phase, is much weaker that the resonance in the red. Its red shift upon approach is much less pronounced than on gold. Also, the decrease in amplitude of the red resonance upon approach is reduced.

Based on these observations, specifically the pronounced red shift seen in Figure 2a, we associate the high-energy resonance with light scattering from the apex of our gold taper, assuming that the red shift is induced by near-field coupling of the apex dipole to its image dipole in the metal. Since the lower-energy resonance does not show such couplings, we tentatively assign it to light scattering from a higher-order mode of the conical gold taper.^58,59^ Only the lowest-order, rotationally symmetric mode of such a taper is a purely evanescent mode, while all higher-order modes are transformed into radiative modes at a finite distance from the apex.^59^ The effective dipole moment of the higher-order mode is essentially oriented parallel to the surface, antiparallel to its image dipole induced in the gold film. Destructive interference therefore results in a decrease of the scattering amplitude upon approach, an effect that is less pronounced on a weakly reflecting dielectric substrate. To characterize the optical properties of apex and shaft mode, we fit σ̅(ω, *d*_*j*_) to a sum of Lorentzian resonances, as discussed in SI section 5. Typical results of such fits for different distances are shown in Figure 2e–g as dotted lines. The extracted response functions of the apex mode are shown in Figure 2h–j. The red shift, line broadening, and decrease in amplitude upon approach are evident.

In nano-optics, such near-field couplings are often rationalized in terms of a phenomenological coupled-dipole model^1,60,61^ which treats the tip as a sphere of radius *a*_0_. Its polarizability is given by the quasistatic response of its dipolar mode. The near-field coupling of this sphere to a planar sample results in an effective polarizability which alters both the amplitude and phase of the light that is scattered from the apex upon approach. To analyze the results in Figure 2, we consider only the longitudinal component of the polarizability, α_*zz*_, pointing along the taper axis. We assume that its frequency dependence can be parametrized in terms of a Lorentz oscillator model^62^ as

2 $$\alpha_{zz}\left(\omega \right)=2\pi \varepsilon_{0}{a_{0}}^{3}\left|\mathrm{FE}\right|e^{i\phi_{\mathrm{FE}}}\left(-\frac{\gamma }{\omega -\omega_{0}+i\gamma }+\frac{\gamma }{\omega +\omega_{0}+i\gamma }\right)$$

where ω_0_ is the resonance frequency and γ is the line width of the apex mode. We introduce a complex-valued field enhancement factor FE, with an amplitude |FE| that gives the enhancement of the near field at the bottom of the sphere (the taper apex) with respect to the incident field. The parameter ϕ_FE_ is introduced to model possible phase shifts between the incident and apex field. Such phase shifts are expected from extensive simulations of the optical field enhancement for such conical metal tapers.^18^ They arise from the finite coupling between the apex near fields and evanescent modes propagating along the taper shaft.^59,63^

As can be seen in Figure 3, the near-field coupling of the apex mode to the gold surface depends sensitively on this phase shift ϕ_FE_. In the simulation, we assume a Lorentzian response with ℏω_0_ = 1.62 eV, ℏγ = 0.12 eV, and |FE| = 18 to mimic the results in Figure 2a,b for large distances. In the absence of a phase shift, ϕ_FE_ = 0, the simulations predict a similar red shift of the apex resonance as observed experimentally. In this case, both the line width of the polarizability and its maximum amplitude remain unchanged upon approach. For small positive values of ϕ_FE_ we observe that the red shift is accompanied by both a line broadening and a reduction in amplitude upon approach, matching the signatures observed in Figure 2. For negative values of ϕ_FE_ we find instead a red shift accompanied by an increase in scattering amplitude and a concomitant line narrowing. Evidently, such negative phase shifts contrast with the experimental observations and are also not expected based on simulations of the field enhancement.^18^ From these Lorentz oscillator simulations, we conclude that we can best describe the results in Figure 2 by assuming a field enhancement factor of the apex mode of ∼18 and a phase shift ϕ_FE_ of about 0.2π. These parameters agree reasonably with those estimated from ref 18 (∼15, ∼0.5π). The simulations also suggest that such a Lorentz oscillator model can describe reasonably well the spectral response of the apex mode. The microscopic origin of the observed distance dependence is readily understood in terms of a perturbative expansion of the effective polarizability in orders of the number of near-field reflections by the sample (see SI section 7). The general trends are captured by considering the interference between the tip polarizability and the first-order change in this polarizability induced by near-field reflection. For ϕ_FE_ = 0, constructive (destructive) interference between components on the low-energy (high-energy) side of the apex resonance results in a pure red shift without changes in line width and amplitude. For ϕ_FE_ = 0.2π, this symmetry is broken, and destructive interference dominates already at energies slightly below the resonance. This lies at the origin of the observed line broadening. Our model suggests that such line broadening is linked with a decrease in scattering amplitude upon approach.

**Figure 3:**
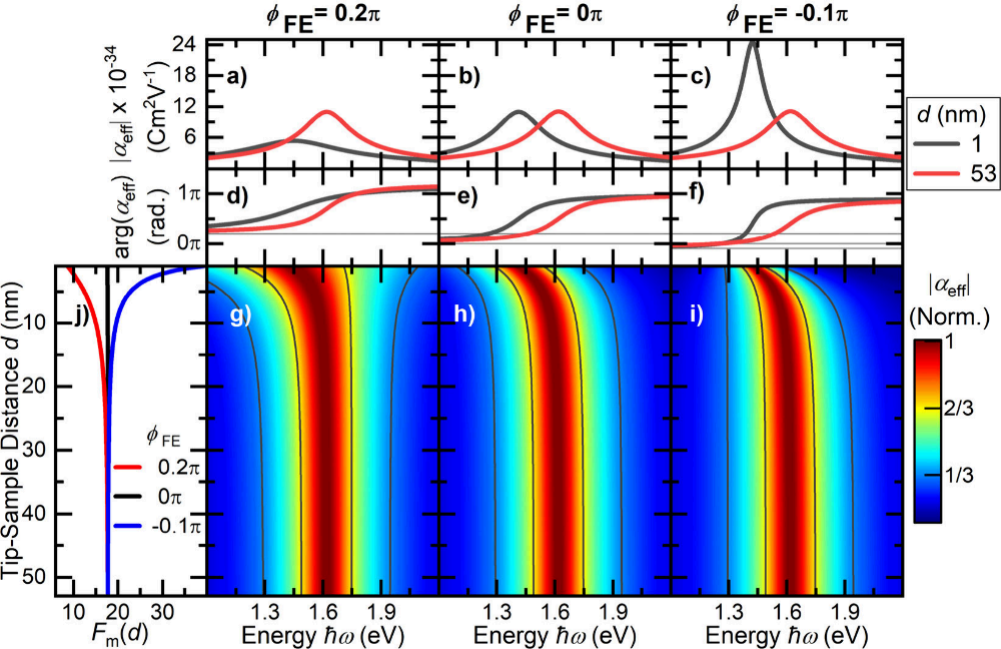
Effective polarizability α_eff_ of a gold tip with 10 nm radius approaching a gold surface as predicted by a Lorentz oscillator point-dipole model. We characterize the longitudinal polarizability of the apex mode by its resonance energy (ℏω_0_ = 1.62 eV), line width (ℏγ = 0.12 eV) and maximum field enhancement (∼18 at ω_0_). The near-field coupling between the apex dipole and its image dipole results in a red shift of α_eff_ upon approach. The effect of the coupling on field enhancement and line width is sensitive to the phase shift between the incident field and near-field at the apex, introduced as ϕ_FE_. (a–c) Amplitude |α_eff_ | for tip–sample distances of 1 nm (black) and 53 nm (red) and for three different values of ϕ_FE_. (d–f) Corresponding spectral phase arg­(α_eff_) of the effective apex dipole polarizability. (g–i) Normalized amplitude |α_eff_| as a function of the tip–sample distance *d* and photon energy ℏω. (j) Distance dependence of the maximum field enhancement *F*_m_(*d*) resulting from the Lorentz oscillator model. The experimentally observed line broadening is predicted for slight positive values of ϕ_FE_ ≈ 0.2π.

With this information, we can now reconstruct the complete near-field dynamics at the apex of our gold taper and, importantly, the effect of near-field coupling on the dynamics. The successful description of the spectral response σ of the apex mode in terms of a Lorentz oscillator model shows that the near-field response *r*(*t*) = $F^{-1}$ ­[σ­(ω)] (where F denotes the Fourier transform) in the time domain is well-described by an exponentially damped sine function *r*(*t*) = Θ­(*t*)­2γ|*F*_m_| sin­(ω_0_ *t* – ϕ_FE_) exp­(−*t*/*T*_2_), where *T*_2_ = 1/γ is the decay time, Θ­(*t*) is the step function, and |*F*_m_| is the maximum field enhancement of the junction for tip–sample distance *d*. This response function gives the time dynamics of the electric field amplitude at the very apex of the gold taper for a fictious δ-pulse excitation. The distance-dependent parameters ω_0_, γ, and |*F*_m_ |/|FE| can be directly taken from the measured spectral response. The complex-valued field enhancement is a material property of the taper that is independent of *d*. Its evaluation needs information that is encoded in the distance dependence of σ. The simulations in Figure 3 show that a field enhancement of FE = 18e^i0.2π^ can characterize the properties of our gold taper reasonably well. Fixing this value provides quantitative access to the near-field electric field amplitude at the apex.

The resulting response functions are shown in Figure 4a,b. The approach to the gold surface not only results in a red shift of ω_0_ but also in a substantial reduction in the decay time *T*_2_ of the near-field response, from 6.1 fs at large *d* to 2.7 fs at 1 nm distance. The distance dependence of the decay time is shown in Figure 4e. The thus-obtained linear near-field response is a property of the coupled tip–sample junction that is independent of the temporal structure of the driving field.

**Figure 4:**
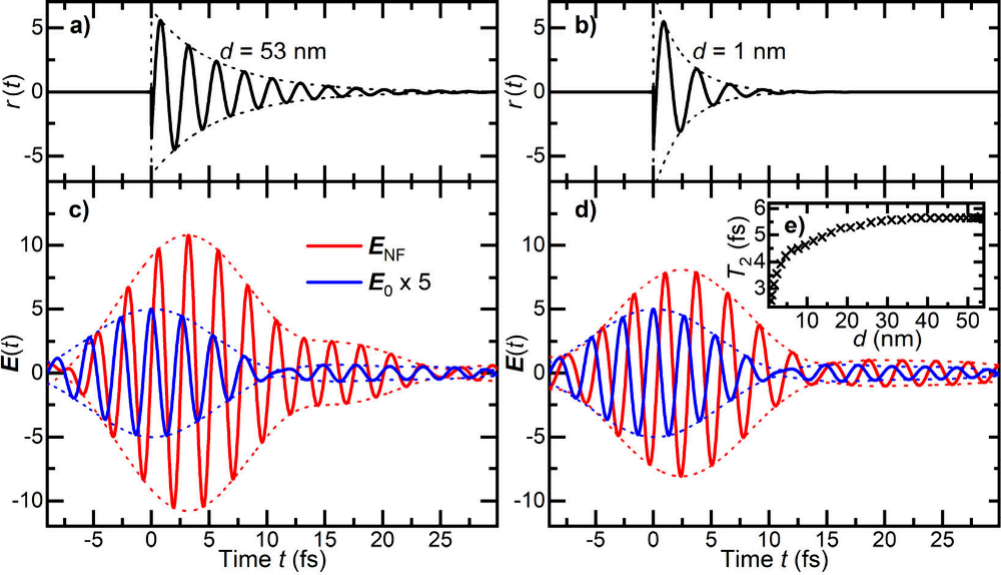
Near-field dynamics in the junction between a gold tip and a gold surface. The time domain response of the tip–sample junction *r*(*t*) linking the incident field **E**_0_ and near-field **E**_NF_ at the apex is given by direct Fourier transform of the distance-dependent spectral response function σ_A_(ω), taking into account the complex-valued field enhancement FE obtained from Figure 3. (a, b) Time-domain response *r*(*t*) for tip–sample distances of (a) 53 nm and (b) 1 nm. The compression of the response function upon approach should be noted. Envelopes are depicted as dotted lines. (c, d) Near-field dynamics **E**_NF_(*t*) (red) at the tip apex for the measured incident field **E**_0_(*t*) (blue). When approaching the gold surface, the near field at the tip apex is temporally compressed. (e) Distance dependence of the decoherence time *T*_2_(*d*) of the near-field response *r*(*t*).

Since we know the time profile *E*_0_(*t*) of our 9 fs incident pulses, we can also obtain the time structure of the local near field *E*_NF_(*t*) at the apex by direct convolution of *E*_0_(*t*) with *r*(*t*). The results are shown in Figure 4c,d. The substantial compression of the few-cycle optical near field when the gap is closed should be noted. For large distances, the near field (red line in Figure 4c) lasts much longer than the incident pulse (blue line in Figure 4c). For small distances, the near-field response is so fast that the near-field and far-field dynamics become very similar. Worthy of note is the phase shift between the near field and far field, reflecting the finite phase of the complex-valued field enhancement.

The results of our analysis for the light scattering from the taper–gold surface junction are summarized in Figure 5a,c. It shows the distance-dependent amplitude (black circles), resonance energy (blue diamonds) and line width (red squares) of the apex mode as obtained from the Lorentz oscillator analysis. The effect of the near-field coupling on these parameters is reasonably well reproduced in our point-dipole model when the complex-valued field enhancement of the tip is taken into account. For our gold taper with an apex radius of ∼10 nm, the near-field coupling effects extend over slightly less than the radius. While the distance dependences of the amplitude and line width are quantitatively described by the point-dipole model, the resonance energy shows an additional longer-range shift that goes beyond the model.

**Figure 5:**
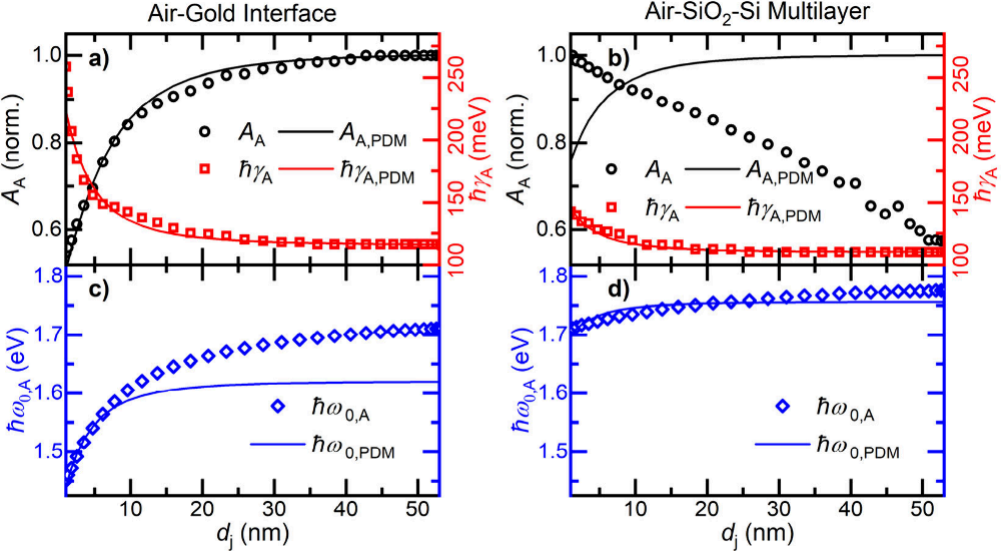
Experimentally measured (open symbols) amplitude (black), line width (red), and resonance energy (blue) of the apex oscillator as a function of the distance between the tip apex and (a, c) the gold surface or (b, d) the SiO_2_/Si substrate. The data are compared to the predictions of a Lorentz oscillator point-dipole model for the apex polarizability. (a, b) Amplitude *A*_A_ (black) and line width ℏγ_A_ (red) of the apex oscillator. (c, d) Corresponding resonance energies ℏω_0,A_ (blue). The oscillator model convincingly reproduces the near-field tip–sample coupling for the gold surface. Taking the same field enhancement, satisfactory agreement of the Lorentz oscillator model is also obtained with the red shift and broadening seen for the dielectric substrate, while the predicted decrease in amplitude in the near-field region is not observed.

To validate these conclusions, we use the same analysis for the SI measurements performed on the dielectric substrate. As shown in Figure S9, we can use the same Lorentz oscillator model to describe the distance-dependent spectral response σ̅(ω, *d*_*j*_) (Figure 2c,d). In this case we need two shaft oscillators with distance-independent resonance energy and line width to accurately reproduce σ̅. The contribution of the apex mode to the scattering signal is much weaker than in the case of the gold film (Figure S11). Also, the effect of near-field coupling on the apex resonance is much reduced, showing a red shift of about 60 meV and a line broadening by about 30 meV upon approach (Figure 5b,d). Both distance dependences are again well-reproduced by the point-dipole model when considering the same complex-valued field enhancement as in the case of the gold surface. In contrast to the predictions of the point-dipole model, the measured amplitude of the apex oscillator shows a slight long-range increase upon approach. We think that this effect, not seen on the gold substrate with strong near-field scattering, mainly reflects residual light scattering from the taper shaft.

In summary, we describe and demonstrate a new technique for probing the electric near-field dynamics in the junction of a scanning probe microscope. Using spectral interferometry with few-cycle pulse excitation, we obtain the amplitude and phase of the light that is scattered from the junction between a gold taper and a gold surface. The near-field coupling between the apex dipole and its image dipole results in a pronounced red shift and line broadening of the apex resonance as the tip–sample gap is closed. We quantify the complex-valued field enhancement at the apex and obtain the local near-field dynamics with subcycle resolution. For this, we use spectral interferometry to extract the higher-order spectral phase of the near field scattered from the apex. From the experimentally observed line shape changes upon approach, we quantitatively extract the phase of the local near-field enhancement at the apex. Together, this provides the desired subcycle resolution of the near-field dynamics.

The parameters deduced for the field enhancement of the gold taper and its phase shift agree reasonably with state-of-the-art finite difference time domain simulations.^18^ Such simulations predict the smooth dependence of these parameters on the tip geometry, specifically the opening angle and apex radius. The measurement technique demonstrated here should allow quantification of the relationship between complex field enhancement and tip geometry in future work. Importantly, the present measurements demonstrate that near-field coupling results in a substantial temporal compression of the few-cycle optical near field for small tip–sample distances. Our results pave the way toward direct time-domain studies of local optical near fields in the visible with substantial importance for probing the local linear and nonlinear response of individual nanostructures with high temporal and spatial resolution.

## Supplementary Material



## Notes

SI-SNOM experimental setup; SEM image of the gold near-field probe; pulse characterization; SI-SNOM spectral response function; transfer function retrieval and data analysis; fitting of the response functions to a Lorentz oscillator model; point-dipole model for the effective polarizability of the apex and shaft dipoles; momentum-dependent generalized Fresnel coefficients; sensitivity of the effective polarizability model (PDF)
