# Supplementary material for: Compressing Few-Cycle Optical Near Fields in the Tip–Sample Junction of a Scanning Probe Microscope
Source: Nano Lett. 2026 Feb 11;26(7):2604–10. doi: 10.1021/acs.nanolett.5c06074 (PMC12947735; doi:10.1021/acs.nanolett.5c06074)
Supplement: Supplementary file 1 [file nl5c06074_si_001.pdf]

Compressing few-cycle optical near fields in the tip-sample junction of a scanning probe microscope

Tom Jehle[1§], Sam S. Nochowitz[1§], Juanmei Duan[1], Christoph Lienau[1,]*

[1]Institut für Physik, Carl von Ossietzky Universität, 26129 Oldenburg, Germany.

[*]Correspondence to: christoph.lienau@uni-oldenburg.de

[§]S.S.N. and T.J. contributed equally to this paper.

1. Spectral interferometry scanning near-field optical microscopy (SI-SNOM): Experimental setup

The experimental setup[1] is schematically depicted in Fig. 1a of the main manuscript. All SI-SNOM experiments are performed with a home-built tapping mode atomic force microscope (AFM).[1-4] A chemically etched gold taper[5] with a radius of curvature of 10 nm (Fig. S1) is used as a near-field probe. The taper is mounted on a quartz tuning fork that is electrically driven to periodically modulate the tip-sample distance at frequency f_t (~ 26 kHz) with an amplitude of 26 nm. The tuning fork current is read out using a lock-in amplifier to monitor the tip-sample distance. The sample is scanned relative to the tip using a three-axis nano positioner (Physik Instrumente, PI-733.3DD).

For the optical experiments, we use light from a 9-fs Titanium-sapphire laser (Fig. S2) operating at a repetition rate of 80 MHz. The laser provides pulses with a spectrum that covers the range from 670 nm to 950 nm. The laser beam is sent into a Michelson interferometer and split into a sample and reference beam using a broad band beam splitter (BS). The beam in the sample arm with a power of a few mW is focused onto the tip apex using a reflective microscope objective (MO, Beck Optronics Solutions, model 5003-000) with a numerical aperture of 0.4. The light that is scattered from the tip-sample junction, i.e., the signal field, is collected using the same objective in a back-reflection geometry. On BS, it is overlapped with the time-delayed beam, the reference field, in the reference arm. The interferometer is slightly unbalanced since the reference beam is reflected off the coated side of BS while the beam that is entering the signal arm enters from the glass side of BS. Both signal and reference are overlapped and coupled into a single mode fiber (Thorlabs PM780 HP) with a core diameter of 5.3 µm and a cut-off wavelength of 710 nm. For the broadband reference field, we reach a coupling efficiency of up to 53%. For estimating the overall coupling efficiency of the light that is scattered from the tip-sample junction, we place the tip in close contact and measure the light before and after the fiber coupler using a power meter. From this, we estimate a fiber coupling efficiency of 10%, For estimating the coupling efficiency for the near-field that is scattered from the tip-sample junction, we replace the power meters by avalanche photodiodes (APD, Hamamatsu C12702-03). The photodiode signals are demodulated at harmonics of f_{tip} and these signals are recorded. We find coupling efficiencies of of 23/36/40% when recording the demodulated APD signal at the 2nd, 3rd and 4th harmonic, respectively. Commonly, the 4th

harmonic signal solely probes optical near-fields that are scattered from the tip-sample junction.

We found that this effective suppression of the omnipresent background scattering from the shaft of the near-field taper is highly important in isolating the spectrum of the light scattered from the apex. The output of the fiber is sent into a low-astigmatism monochromator (Princeton Instruments, IsoPlane-160) and then recorded with a fast line scan camera (e2V AViiVA EM4 with 512 pixels) operating at a read-out frequency of 217 kHz. This enables the acquisition of individual spectral interferograms (SI) with an acquisition time of 4.6 μs. This acquisition time is shorter than the tip oscillation period of ~40 μs and thus enables us to record spectra at different tip-sample distances. Also, the short acquisition time suppresses any mechanical motion of the interferometer and therefore ensures phase stability of the SI.

The camera offers a line repetition rate of 217 kHz (4.6 μs) and has 12 bit resolution (maximally 4095 counts per pixel). Experimentally, the best results are obtained when the reference field is so strong that it almost saturates the camera. This requires a reference power of ~ 4 μW. The dynamic range of the camera is ~ 3000:1, implying that the signal field can be approximately 3000 times weaker than the reference field. This sets a lower limit for the signal power of ~ 4 μW / $(3000)^2$ ~ 0.4 pW. We have performed the measurement with signal powers of ~ 2 nW, giving a modulation contrast of the SI of ~ 10%.

The camera sensitivity of ~ 60 photons/count and the maximum of 4095 counts per pixel implies that up to 240.000 photons can arrive at a single pixel on the line camera within the 4.6 μs exposure time.

Experimentally, we record up to ≈ 32000 spectra at each sample position during a total acquisition time of 0.15 seconds. This configuration provides a stable and efficient platform for measuring few-cycle field dynamics in the tip-sample junction with nanometric spatial resolution.

2. SEM image of the gold near-field probe

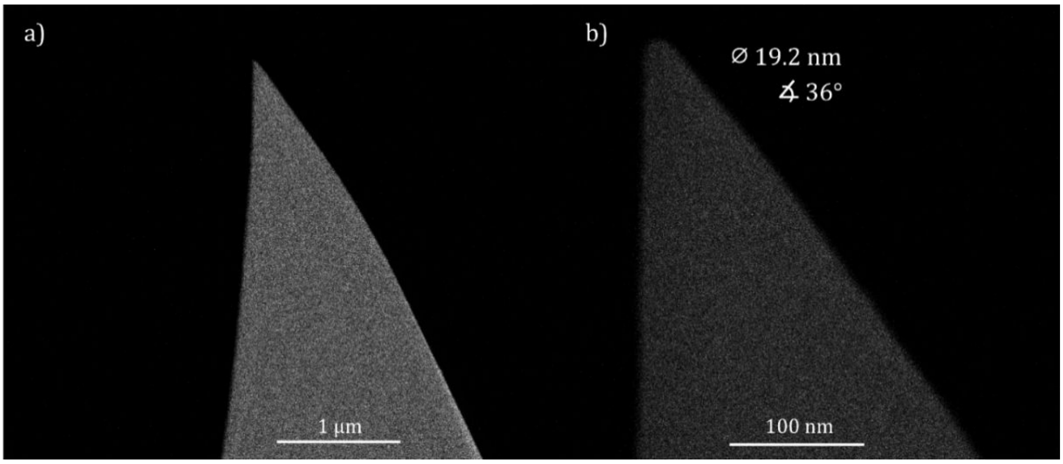

Figure S1: Scanning electron microscope image of the gold tip that is used as a probe in the experiments. The tip has an opening angle of 36° and an apex diameter of 19.2 nm. The tip is electrochemically etched in hydrochloric acid from annealed 99.99+% pure gold wire (Advent Research Materials AU5173, ≈ 20 µm grain size, 125 µm diameter). Quasistatic calculations suggest that the lateral confinement of the near-field intensity in a nanoscale gap scales as $w \approx 1.6\sqrt{Rd}$, where R is the apex radius and d the tip-sample distance[6]. We therefore that such tips shall allow to reach lateral near-field confinements down to 5 or even below.

3. Pulse characterization

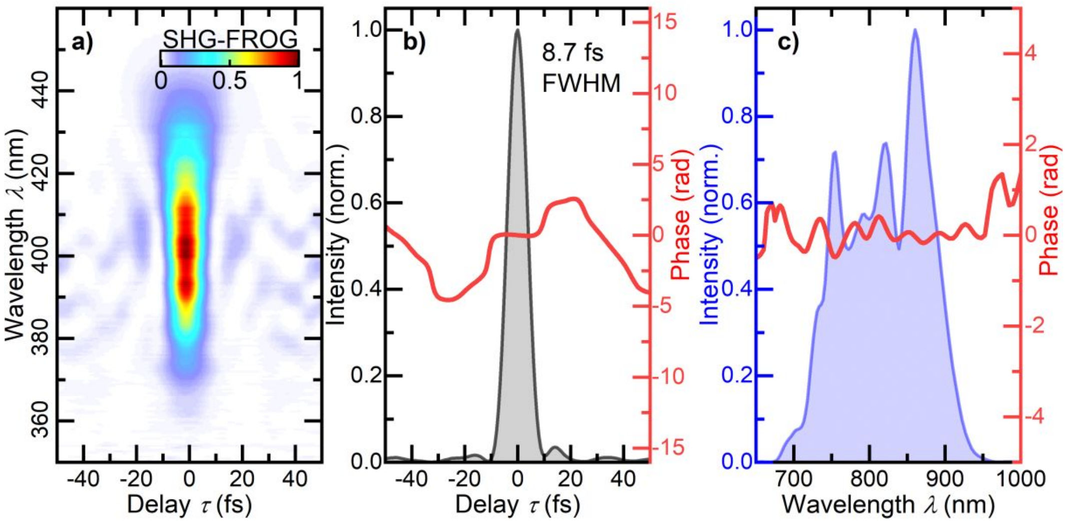

Figure S2: Second Harmonic Generation Frequency-Resolved Optical Gating (SHG-FROG) of typical laser pulses used in the experiment.[7] **a)** SHG-FROG trace obtained from a Interferometric, frequency-resolved autocorrelation (IFRAC) measurement performed using a nonlinear a-BBO crystal placed at the tip position in the focus of the all-reflective microscope objective in Fig. 1.[8, 9] For the IFRAC measurements, we have used a phase-locked pulse pair, generated in an external Mach-Zehnder interferometer. The second harmonic emitted from the BBO crystal has been collected using the same objective, spectrally dispersed in a monochromator and recorded as a function of the interpulse delay. We have used established phase-retrieval techniques to reconstruct amplitude and spectral phase of the laser pulses. **b)** Temporal intensity profile and temporal phase of the pulse retrieved from the IFRAC measurement. **c)** Intensity spectrum and spectral phase of the retrieved pulse. The retrieved pulse duration is 8.7 fs (full width at half maximum of the intensity profile). The remaining sinusoidal spectral phase is caused by the Novanta DCM7 chirped mirror pair used for pre-compensation of the pulse.

4. Spectral interferometry scanning near-field optical microscopy (SI-SNOM): Spectral response function

This section introduces the transfer function σ that connects signal field $\boldsymbol{E}_{\mathbf{S}}$ and the laser field $\boldsymbol{E}_{\mathbf{0}}$ that enters the beam splitter (BS) of an asymmetric, unbalanced Michelson Interferometer in the SI-SNOM experiments (Fig. 1). At BS, the incident field is split into a field that enters

the signal arm and a field that enters the reference arm. The field in the signal arm is focused onto the taper and generates the signal field $\boldsymbol{E}_{\mathbf{S}}$ that is emitted from the tip-sample junction. The field that enters the second arm of the interferometer generates a time-delayed reference field $\boldsymbol{E}_{\mathbf{R}}$. Both fields are spatially overlapped at BS and are focused into the core of a single mode fiber (SMF).

For further discussion, we consider, for simplicity, that a monochromatic, plane-wave incident field $\boldsymbol{E}_{\mathbf{0}}(\boldsymbol{r},t)$, propagating along the x-direction, enters into the interferometer. We assume a field that is linearly polarized along $\hat{\mathbf{e}}_{\mathbf{z}}$ and is defined by its angular frequency ω, wave vector $\boldsymbol{k}_{\mathbf{0}} = k_0\hat{\mathbf{e}}_{\mathbf{x}}$ with unit vector $\hat{\mathbf{e}}_{\mathbf{x}}$, and amplitude E_0

$$\boldsymbol{E}_{\mathbf{0}}(\boldsymbol{r},t) = E_0\hat{\mathbf{e}}_{\mathbf{z}}e^{i\boldsymbol{k}_{\mathbf{0}}\cdot\boldsymbol{r}}e^{-i\omega t}(1)$$

Denoting the time delay in the reference arm by τ_0 and the complex-valued fiber coupling coefficient of the reference field as $t_R(\omega)$, the reference field can be written as $\boldsymbol{E}_{\mathbf{R}} = E_{\mathrm{R}}\hat{\mathbf{e}}_{\mathbf{z}} = t_{\mathrm{R}}(\omega)e^{i\omega\tau_0}\boldsymbol{E}_{\mathbf{0}}$. Since the reference field is linearly polarized along the z-axis, SI-SNOM measures the transfer function that connects the z-components of both fields, $\sigma = E_{\mathrm{S}}/E_0$ with $E_{\mathrm{S}} = \boldsymbol{E}_{\mathbf{S}} \cdot \hat{\mathbf{e}}_{\mathbf{z}}$.

To discuss the physical meaning of this transfer function, we assume that the signal field at the detector position is the sum of two scattering contributions. The first contribution is (i) the desired "near-field" contribution $\boldsymbol{E}_{\mathbf{NF}} = \overleftrightarrow{\mathbf{M}}_{\mathbf{NF}} \cdot \boldsymbol{E}_{\mathbf{0}}$ that is scattered from the very apex of the tip, or, more precisely from the tip-sample junction. In addition, (ii) a background field $\boldsymbol{E}_{\mathbf{B}} = \overleftrightarrow{\mathbf{M}}_{\mathbf{B}} \cdot \boldsymbol{E}_{\mathbf{0}}$ is detected that is scattered from the shaft of the gold taper. Therefore, the total signal field is expressed as:

$$\boldsymbol{E}_{\mathbf{S}} = \left(\overleftrightarrow{\mathbf{M}}_{\mathbf{NF}} + \overleftrightarrow{\mathbf{M}}_{\mathbf{B}}\right) \cdot \boldsymbol{E}_{\mathbf{0}}(2)$$

In this plane-wave approximation, near-field and background can be connected to the incident field by a sequence of transfer matrices[1]

$$\overleftrightarrow{\mathbf{M}}_{\mathbf{NF}}(d) = t_{\mathrm{NF}}\overleftrightarrow{\mathbf{M}}_{\mathbf{out}}\overleftrightarrow{\mathbf{G}}_{\mathbf{0}}\overleftrightarrow{\boldsymbol{\alpha}}_{\mathbf{eff}}\overleftrightarrow{\mathbf{M}}_{\mathbf{in}} (3)$$

and

$$\overleftrightarrow{\mathbf{M}}_{\mathbf{B}} = t_{\mathrm{B}}\overleftrightarrow{\mathbf{M}}_{\mathbf{out}}\,\overleftrightarrow{\mathbf{M}}_{\mathbf{B'}}\,\overleftrightarrow{\mathbf{M}}_{\mathbf{in}} (4)$$

The transfer matrix $\overleftrightarrow{\mathbf{M}}_{\mathbf{NF}}$ consist of three terms: (i) The matrix $\overleftrightarrow{\mathbf{M}}_{\mathbf{in}}$ describes the propagation of the incident field to the tip-sample junction.[1] This generates the field $\boldsymbol{E}_{\mathbf{in}} = \overleftrightarrow{\mathbf{M}}_{\mathbf{in}} \cdot \boldsymbol{E}_{\mathbf{0}}$ that interacts with the tip. (ii) The interaction of $\boldsymbol{E}_{\mathbf{in}}$ with the apex of the near-field taper. This interaction is described in point-dipole approximation, generating a apex dipole moment $\boldsymbol{p}_{\mathbf{A}}^{(\mathbf{0})} = \overleftrightarrow{\boldsymbol{\alpha}}_{\mathbf{eff}}\boldsymbol{E}_{\mathbf{in}}$, where $\overleftrightarrow{\boldsymbol{\alpha}}_{\mathbf{eff}}(\omega,d)$ is an effective, frequency-dependent dipole polarizability of the coupled tip-sample system.[10-12] This effective polarizability depends sensitively on the tip-sample coupling and, thus, on the tip-sample distance d. Our model for $\overleftrightarrow{\boldsymbol{\alpha}}_{\mathbf{eff}}$ will be discussed in Section 7. (iii) The emission of far-field radiation by the apex dipole and its coupling into the single mode fiber $\boldsymbol{E}_{\mathbf{NF}} = t_{\mathrm{NF}}\,\overleftrightarrow{\mathbf{M}}_{\mathbf{out}}\,\overleftrightarrow{\mathbf{G}}_{\mathbf{0}}\,\boldsymbol{p}_{\mathbf{A}}^{(\mathbf{0})}$. Here, $\overleftrightarrow{\mathbf{G}}_{\mathbf{0}}$ is the far-field dyadic Green function for the apex dipole[13] and the collection of the emitted light and its coupling into the fiber is described by the product of a coupling efficiency t_{NF} and a transfer matrix $\overleftrightarrow{\mathbf{M}}_{\mathbf{out}}$.

In the same way, we decompose $\overleftrightarrow{\mathbf{M}}_{\mathbf{B}}$ into a product of $\overleftrightarrow{\mathbf{M}}_{\mathbf{in}}$, a yet undefined transfer matrix $\overleftrightarrow{\mathbf{M}}_{\mathbf{B'}}$ $\overleftrightarrow{\mathbf{M}}_{\mathbf{out}}$ and a fiber coupling efficiency t_{B}.

For our linearly polarized reference field, the transfer function $\sigma(\omega,d) = \sigma_{\mathrm{NF}}(\omega,d) + \sigma_{\mathrm{B}}(\omega)$ is then given by the scalar functions:[1]

$$\sigma_{\mathrm{NF}}(\omega,d) = \left(\overleftrightarrow{\mathbf{M}}_{\mathbf{NF}}(\omega,d)\right)_{\mathrm{zz}} (5)$$

$$\sigma_{\mathrm{B}}(\omega) = \left(\overleftrightarrow{\mathbf{M}}_{\mathbf{B}}(\omega)\right)_{\mathrm{zz}} (6)$$

5. Transfer function retrieval and data analysis

This section explains how we extract distance-dependent response functions from the recorded spectral interferograms (SI). Experimentally, we measure a series of N_{k} ~ 32.000 SIs[14, 15] $SI(\omega, t_{\mathrm{k}})$ at time points $t_{\mathrm{k}} = (k-1)\Delta t$ with a time step of $\Delta t = 4.6$ µs. Each SI is integrated for 2.2 µs. Using the complex-valued transfer function

$$\sigma(\omega) = |\sigma(\omega)| \exp(i\varphi(\omega)) = E_{\mathrm{S}}(\omega)/E_0(\omega) (7)$$

and $t_{\mathrm{R}}(\omega) = |t_{\mathrm{R}}(\omega)| \exp\big(i\varphi_{\mathrm{R}}(\omega)\big)$, the SIs can be written as

$$SI(\omega, t_{\mathrm{k}}) \approx I_0(\omega) \left(|t_{\mathrm{R}}|^2 + |\sigma(\omega, t_{\mathrm{k}})|^2 + 2|\sigma(\omega, t_{\mathrm{k}})|\,|t_{\mathrm{R}}| \cos(\omega\tau_0 - \varphi_{\mathrm{R}} + \varphi(\omega, t_{\mathrm{k}}))\right). (8)$$

Here, $I_0(\omega) = |E_0(\omega)|^2$ is the intensity spectrum of the reference field and τ_0 is the time delay of -340 fs that is introduced in the reference arm. After Fourier transformation to the time domain, the third term in Eq. (8) will give rise to side peaks centered around $\pm\tau_0$. Each of these side peaks can be selected and back-transformed into the spectral domain. Their analysis provides the desired spectral amplitude and phase of $\sigma(\omega)$,[3, 16] as shown in more detail below. We associate each time step t_{k} with a tip-sample distance as:

$$d(t_{\mathrm{k}}) = d_0 + \Delta d \sin(\omega_{\mathrm{m}} t_{\mathrm{k}} + \phi_{\mathrm{m}}) (9)$$

Here, $\omega_{\mathrm{m}} = 2\pi f_{\mathrm{t}}$ is the angular tip modulation frequency, $\Delta d = 26$ nm the tapping amplitude and ϕ_{m} a phase offset. The amplitude d_0 is set to 27 nm to ensure a closest tip-sample distance of ≈ 1 nm. A small selection of the measured SIs is shown in Fig. S3a. The tip-sample distance $d(t_{\mathrm{k}})$ for each SI is shown in Fig. S3b as a blue square. These tip-sample distances are deduced by fitting the amplitude of $SI(\hbar\omega = 1.45\ \mathrm{eV}, t_{\mathrm{k}})$ at a fixed photon energy to Eq. (9) and by

extracting the unknown phase ϕ_{m} from this fit. A representative $SI(\omega,t_5)$ at time $t_5 = 18.4$ μs is depicted in Fig. S3c.

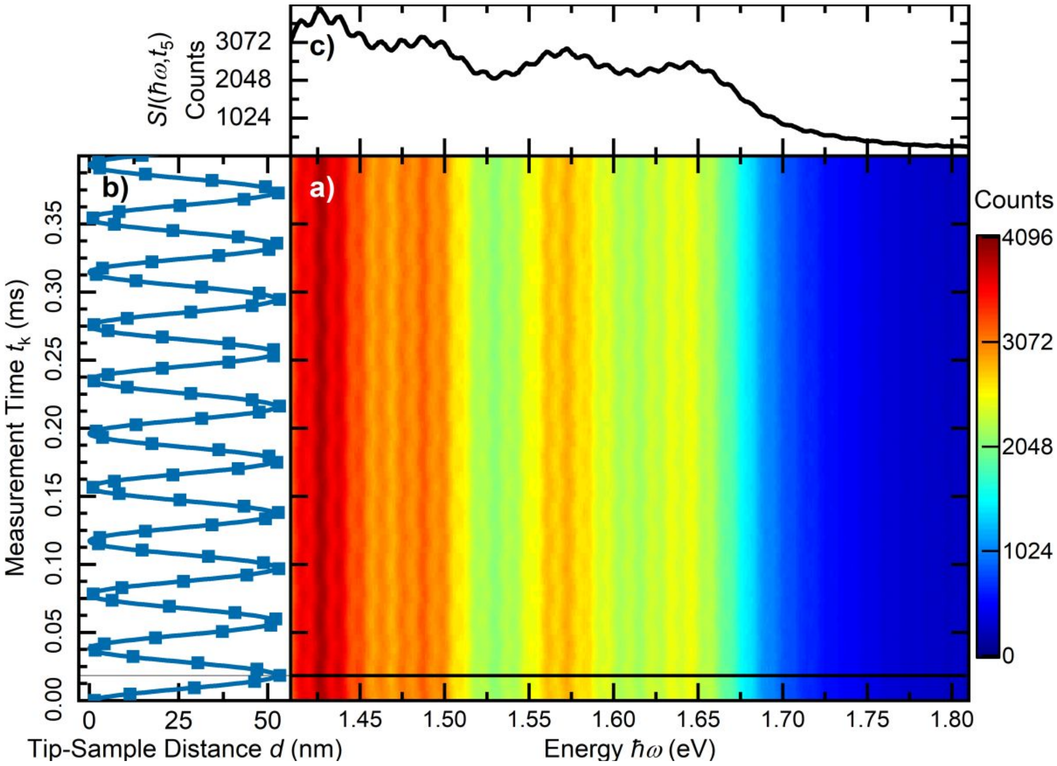

Figure S3: Sequence of spectral interferograms (SI) recorded from the junction between an etched gold taper gap and a gold surface. **a)** SIs recorded while periodically modulating the tip-sample distance. The time step between subsequent SIs is 4.6 μs. The spectra are plotted as a function of photon energy $\hbar\omega$. **b)** Tip-sample distances d associated with each interferogram (blue squares). **c)** Exemplary SI, recorded at $t_5 = 18.4$ μs. The fringe contrast of ~ 10% implies that the maximum signal field amplitude is ~ 40 times weaker than the reference field amplitude.

In addition, a series of spectra is recorded with either the signal arm blocked ($I_{\mathrm{R}}(\omega,t_{\mathrm{k}})$) or the reference arm blocked ($I_{\mathrm{S}}(\omega,t_{\mathrm{k}})$). From these spectra, we deduce an average reference spectrum $\bar{I}_{\mathrm{R}}(\omega) = \frac{1}{N_{\mathrm{k}}}\sum_{k=1}^{N_{\mathrm{k}}} I_{\mathrm{R}}(\omega,t_{\mathrm{k}})$ and an average signal spectrum $\bar{I}_{\mathrm{S}}(\omega) = \frac{1}{N_{\mathrm{k}}}\sum_{k=1}^{N_{\mathrm{k}}} I_{\mathrm{S}}(\omega,t_{\mathrm{k}})$. For transforming these spectra into the time, the following definitions of the Fourier transform are used:[17] $S(\omega) = \mathcal{F}\left[s(t)\right] = \int_{-\infty}^{\infty} s(t)e^{i\omega t}dt$ and $s(t) = \mathcal{F}^{-1}\left[S(\omega)\right] = \frac{1}{2\pi}\int_{-\infty}^{\infty} S(\omega)e^{-i\omega t}d\omega$.

This gives the time domain signals $si(t,t_\mathrm{k}) = \mathcal{F}^{-1}[SI(\omega,t_\mathrm{k})]$, $\bar{i}_\mathrm{R}(t) = \mathcal{F}^{-1}[\bar{\mathrm{I}}_\mathrm{R}(\omega_\mathrm{i})]$ and $\bar{i}_\mathrm{S}(t) = \mathcal{F}^{-1}[\bar{\mathrm{I}}_\mathrm{S}(\omega_\mathrm{i})]$. Around time zero, the DC part of each SI in the time domain, $si(t,t_\mathrm{k})$, is approximately given by the sum $\bar{i}_\mathrm{R}(t) + \bar{i}_\mathrm{S}(t)$ (Fig. S4a). We therefore approximate each time-domain SI in the range from -62 fs < t < 62 fs as

$$si(t,t_\mathrm{k}) \approx u_\mathrm{k}\left(\bar{i}_\mathrm{S}(t) + \bar{i}_\mathrm{R}(t)\right)(10)$$

The real-valued scaling factors u_k are evaluated using least squares fitting. The DC part of each SI is removed using

$$si_\mathrm{c}(t,t_\mathrm{k}) = si(t,t_\mathrm{k}) - u_\mathrm{k}\left(\bar{i}_\mathrm{S}(t) + \bar{i}_\mathrm{R}(t)\right)(11)$$

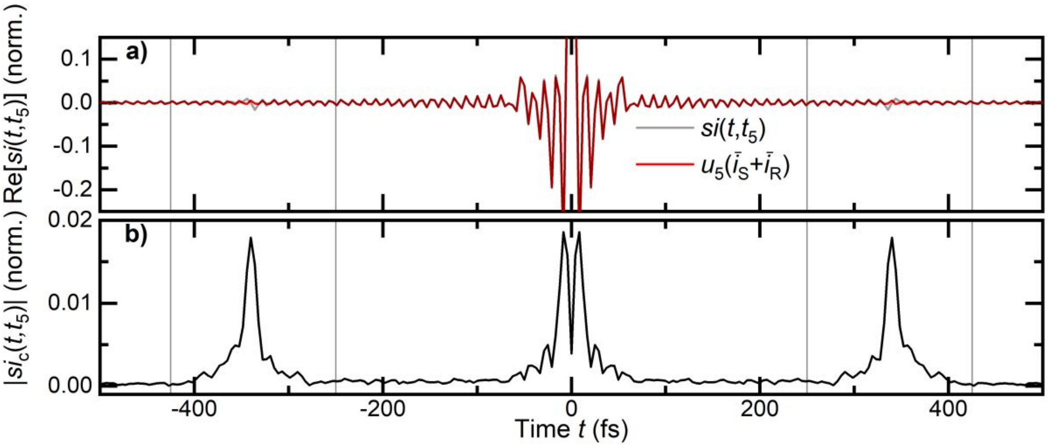

Figure S4: DC-part removal from SI. **a)** Normalized real part of an exemplary time-domain SI, $si(t,t_5)$ (grey line), and fit $u_5\left(\bar{i}_\mathrm{S}(t)+\bar{i}_\mathrm{R}(t)\right)$ (red line) to the time-domain signals obtained from the signal and reference arm spectra. **b)** Amplitude of the time-domain SI, $si_\mathrm{c}(t,t_5)$ after removal of the DC part. Time domain oscillations resulting from the Fourier transform of SIs are effectively suppressed.

An example of the absolute value of the resulting corrected time-domain SIs is shown in Fig. S4b. It is evident that the pronounced DC part of the original SI is mostly removed.

In the next step, we select the AC part of the SI that is centered around $t =$ 340 fs by multiplying the SI with a super-gaussian filter $f(t) = \exp\left(-\left(\frac{(t-t_c)^2}{2t_\mathrm{w}^2}\right)^d\right)$ (Fig. S5a, red line)

$$si_+(t,t_k) = si_c(t,t_k)f(t)(12)$$

This filtering isolates the cross-term contribution around the delay τ_0, effectively separating it from the DC background. For the filtering, we choose $d = 8$, $t_W = \Delta t_F\left(2\sqrt[2d]{2^d \ln(2)}\right)$, $\Delta t_F = 520$ fs and $t_c = 420$ fs. Here, Δt_F is the full width at half maximum (FWHM) of the filter and t_c is the filter center. Both are chosen as large as possible to optimize the spectral resolution of the resulting response function.

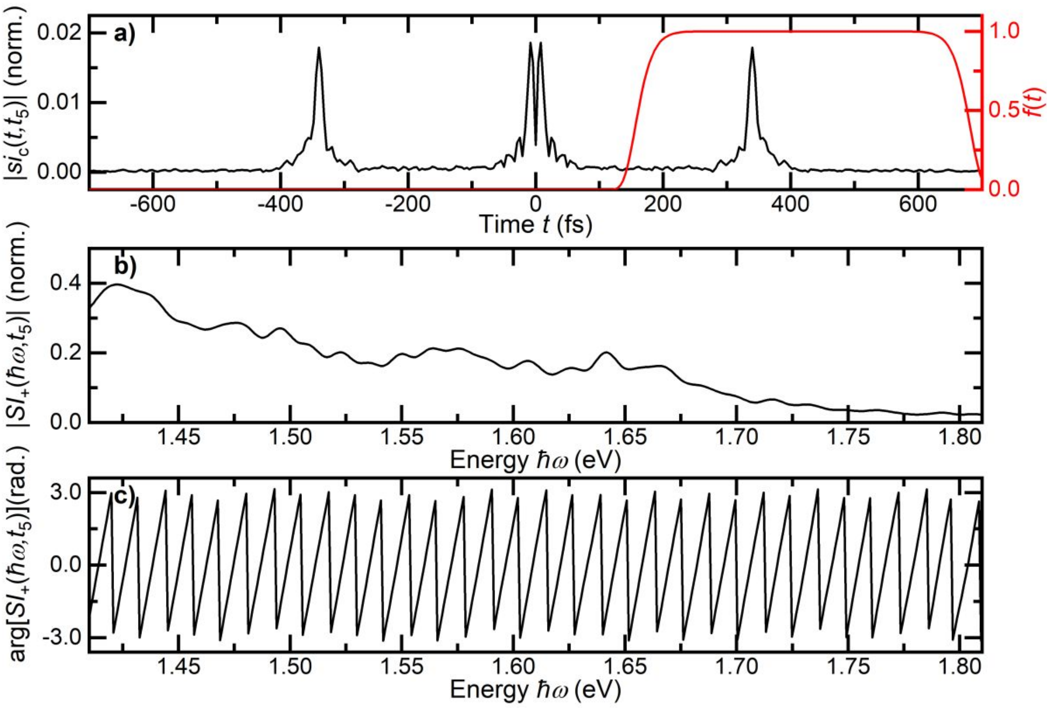

Figure S5: Time domain filtering of the SIs. **a)** Amplitude of the time domain profile of a selected SI after DC-part subtraction, $|si_c(t,t_5)|$ (black line), and filter function $f(t)$ (red line). **b)** Amplitude $|SI_+(\hbar\omega,t_5)|$ (black line) and **c)** spectral phase $\arg\left(SI_+(\hbar\omega,t_5)\right)$ (red line) of the filtered SI in the frequency domain. The spectral phase is mainly dominated by the phase $\omega\,\tau_0$ introduced by the time delay in the reference arm.

The SIs are transformed back into the frequency domain, giving

$$SI_+(\omega,t_k) = \mathcal{F}\left[si_+(t,t_k)\right](13)$$

Amplitude and spectral phase of $SI_+(\hbar\omega,t_5)$ are shown as examples in Fig. S5b and c, respectively. Comparison to Eq. (8) shows that

$$SI_+(\omega,t_\mathrm{k}) = I_0(\omega)\,|\sigma(\omega,t_\mathrm{k})|\,|t_\mathrm{R}(\omega)|\,\exp\left(i\left(\omega\tau_0 - \varphi_\mathrm{R} + \varphi(\omega,t_\mathrm{k})\right)\right)(14)$$

Thus, amplitude and spectral phase of the transfer function can be deduced from SI_+ as

$$|\sigma(\omega,t_\mathrm{k})| = |SI_+(\omega,t_\mathrm{k})|\,|t_\mathrm{R}(\omega)|/\bar{I}_\mathrm{R}(\omega),\ (15)$$

$$\varphi(\omega,t_\mathrm{k}) = \left(\arg\left(SI_+(\omega,t_\mathrm{k})\right) - \omega\tau_0 + \varphi_\mathrm{R}(\omega)\right)(16)$$

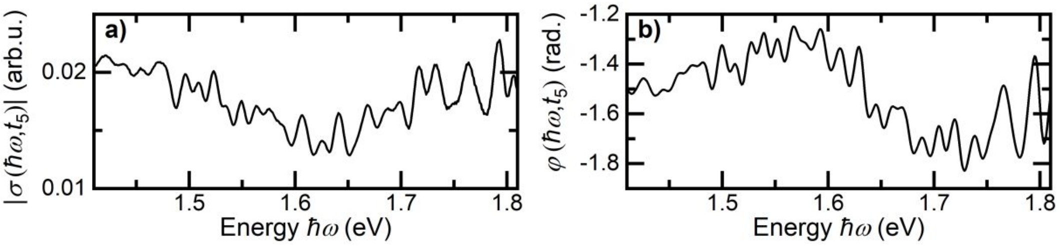

Figure S6: Transfer function $\sigma(\omega)$ retrieved from a single spectral interferogram. **a)** Amplitude $|\sigma(\omega,t_5)|$ and **b)** spectral phase $\varphi(\omega,t_5)$ retrieved from the spectral filtering of the positive AC peak $si_+(t,t_5)$ recorded at time $t_5 =$ 18.4 µs.

In Eqs. (15) and (16), the unbalanced part of the interferometer is introduced via the transmission coefficient $t_\mathrm{R}(\omega)$. Experimentally, we find that $t_\mathrm{R}(\omega)$ partly comes from the asymmetric transmission of signal and reference beam through the beam splitter BS (Thorlabs, UFBS5050). We have calibrated the transmission coefficient $t_\mathrm{R}(\omega)$ experimentally and use this calibration curve when extracting the response function $\sigma(\omega)$ from SI_+ with the help of Eqs. (15) and (16). The retrieved amplitude $|\sigma(\omega,t_5)|$ and spectral phase $\varphi(\omega,t_5)$ are shown in Fig. S6a,b respectively. The SI_- signal, deduced from the AC-peak si_- carries the same information $|\sigma(\omega,t_\mathrm{k})|$ and $\varphi(\omega,t_\mathrm{k}) = -\left(\arg\left(SI_-(\omega,t_\mathrm{k})\right) + \omega\tau_0 + \varphi_\mathrm{R}(\omega)\right)$.

We sort the response functions $\sigma(\omega,t_\mathrm{k})$ into $N_\mathrm{B} =$ 32 distance bins. When performing this sorting, it is important to differentiate between small and large tip-sample distances.

Experimentally, we distinguish the two cases by comparing measurements with different tapping amplitudes. Measurements with reduced tapping amplitude lack spectra for the largest tip-sample distance. A comparison of the two measurements thus provided the desired information. To optimize the signal-to-noise ratio, we sort 1024 functions into each bin. Averaging over these gives distance-dependent response functions $\bar{\sigma}(\omega,d_{\mathrm{j}})$ with $j = 1,...,N_{\mathrm{B}}$. The resulting non-equidistant axis starts at $d_1 = 2$ nm and ends at $d_{32} = 52$ nm. The average bin width is 1.7 nm.

6. Fitting of the response functions to a Lorentz oscillator model

We employ a phenomenological Lorentz oscillator model to parametrize the experimentally measured distance-dependent response functions $\bar{\sigma}(\omega,d_{\mathrm{j}}) = |\bar{\sigma}(\omega,d_{\mathrm{j}})| \exp\left(i\bar{\varphi}(\omega,d_{\mathrm{j}})\right)$ recorded when approaching the tip to a gold surface or a thin SiO_2 film on a silicon substrate. The aim is to extract the physical parameters that characterize the apex and the shaft mode of our near-field probe. For this, we use a model of a minimum number of Lorentzian oscillators. In the case of the gold surface, we restrict this model to two oscillators, one describing the apex mode and another one for the shaft mode. In the case of the dielectric film, we find that it is necessary to include two different oscillators to describe the response of the shaft. Each Lorentzian oscillator is characterized by a normalized lineshape function

$$L(\omega) = -\frac{\gamma}{\omega - \omega_0 + i\gamma} \quad (17)$$

with resonance frequency ω_0 and linewidth $\gamma = 1/T_2$ (T_2: dephasing time). We allow for a finite relative phase $\Delta\phi$ between tip and shaft fields and assume that all fields are phase shifted by $\phi(\omega) = \phi_0 + \phi_1\omega$ with respect to the reference field.

For the gold surface, we take the apex response as $\sigma_{\mathrm{A}}(\omega) = A_{\mathrm{A}}L_{\mathrm{A}}(\omega)$ and the shaft response as $\sigma_{\mathrm{S}}(\omega) = A_{\mathrm{S}}L_{\mathrm{S}}(\omega)e^{i\Delta\phi}$ and obtain

$$\sigma_{\mathrm{M}}(\omega) = \left(\sigma_{\mathrm{A}}(\omega) + \sigma_{\mathrm{S}}(\omega)\right)e^{i(\phi_0+\phi_1\omega)} \quad (18)$$

For the dielectric film we use

$$\sigma_{\mathrm{M}}(\omega) = \left(\sigma_{\mathrm{A}}(\omega) + \sum_{\mathrm{i}=1}^{2} \sigma_{\mathrm{S,i}}(\omega)\right)e^{i(\phi_0+\phi_1\omega)} \quad (19)$$

We simplify the fitting of the response functions by choosing distance-independent resonance energies and linewidths for the shaft oscillators. Also, the relative phases $\Delta\phi$ are kept distance-independent. All oscillator amplitudes A are taken as real-valued, positive numbers.

Amplitude and spectral phase of $\sigma_{\mathrm{M}}(\omega, d_{\mathrm{j}})$ that are obtained from a fit to the response functions for the gold surface are shown in Fig. S7c,d, respectively. The convincing agreement between data and model is demonstrated by plotting the error $|\bar{\sigma}_{\mathrm{Au}} - \sigma_{\mathrm{M}}|$ in Fig. S7e. The parameters that are extracted from these fits are shown, for the gold surface, in Fig. S8.

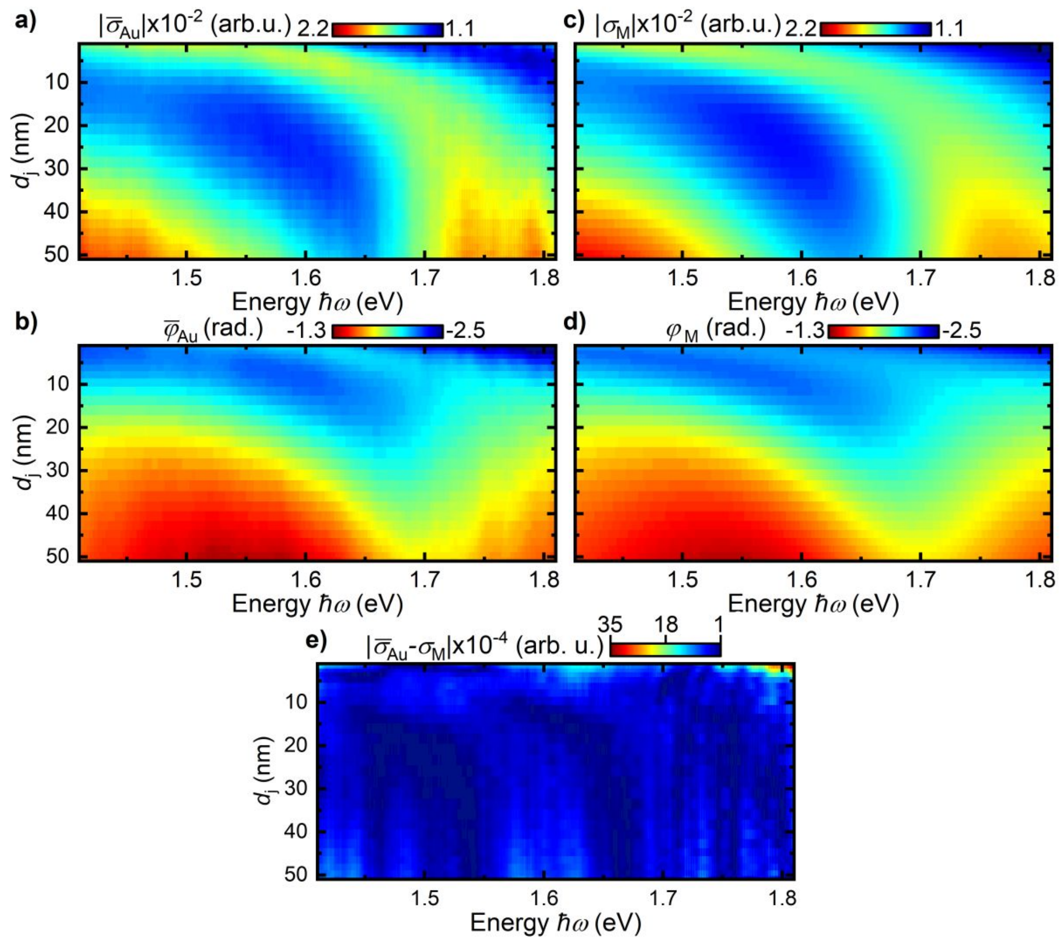

Figure S7: Response function $\bar{\sigma}(d_\mathrm{j},\hbar\omega)$ recorded when approaching the tip to a gold surface and fit to a two-oscillator model. $\bar{\sigma}$ is plotted as a function of the tip-sample distance d_j and the photon energy $\hbar\omega$. **a,b)** Amplitude $|\bar{\sigma}_\mathrm{Au}|$ (**a**) and spectral phase $\bar{\varphi}_\mathrm{Au}$ (**b**) of $\bar{\sigma}$. **c,d)** Amplitude $|\sigma_\mathrm{M}|$ (**c**) and spectral phase φ_M (**d**) obtained from a fit to the data. **e)** Deviation of the fit from the measured response function $|\bar{\sigma}_\mathrm{Au} - \sigma_\mathrm{M}|$.

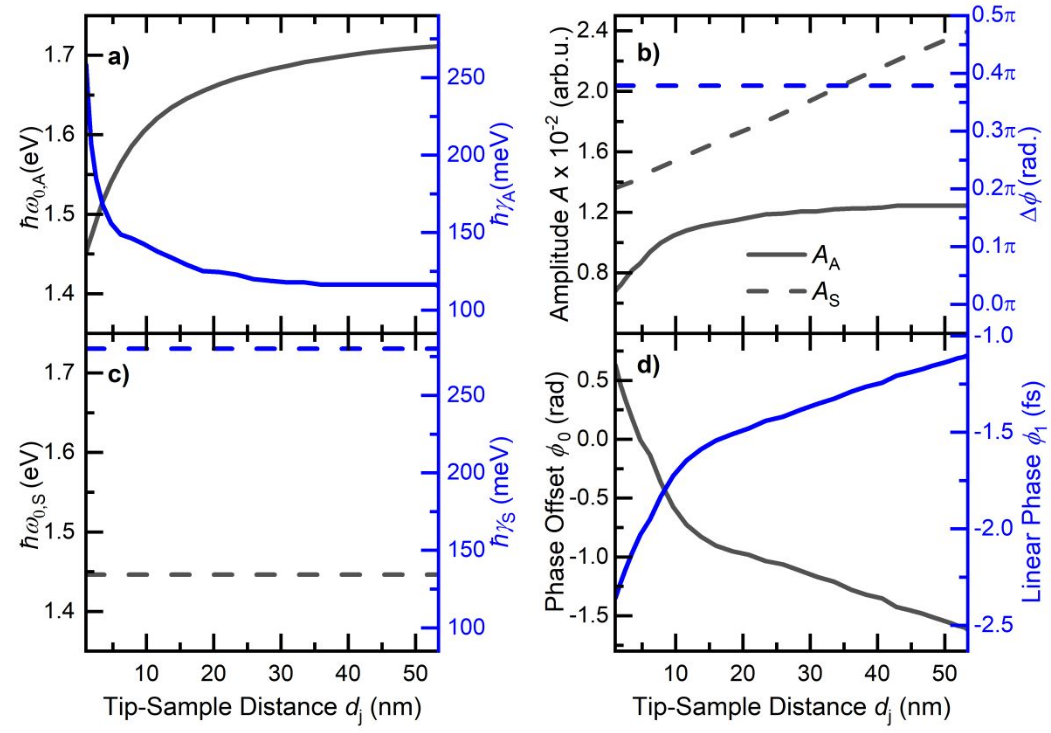

Figure S8: Resonance energy, linewidth and amplitude of apex and shaft modes as obtained from a fit to the data for the gold surface in Fig. S7. **a)** Distance dependent resonance energy $\hbar\omega_{0,\mathrm{A}}$ (black line) and linewidth $\hbar\gamma_{\mathrm{A}}$ (blue line) of the apex mode, showing the pronounced redshift and line broadening upon approach to the gold surface. **b)** Amplitudes A_{A} (black solid line) and A_{S} (black dashed line) of apex and shaft mode, respectively. The relative phase shift $\Delta\phi$ between both oscillators is depicted as a dashed blue line. **c)** Resonance energy $\hbar\omega_{0,\mathrm{S}}$ (black dashed line) and linewidth $\hbar\gamma_{\mathrm{S}}$ (blue dashed line) of the shaft mode. **d)** Phase offset ϕ_0 (black line) and linear phase ϕ_1 (blue line) of the field emitted from the tip-sample junction.

Amplitude and spectral phase of $\sigma_{\mathrm{M}}(\omega, d_{\mathrm{j}})$ that are obtained from a fit to the response functions for the SiO_2/Si substrate are given in Fig. S9c,d, respectively. Again, we reach convincing agreement between data and model, as evidenced by plotting the error $|\bar{\sigma}_{\mathrm{Sub}} - \sigma_{\mathrm{M}}|$ in Fig. S9e. The parameters that are extracted from these fits are shown in Fig. S10.

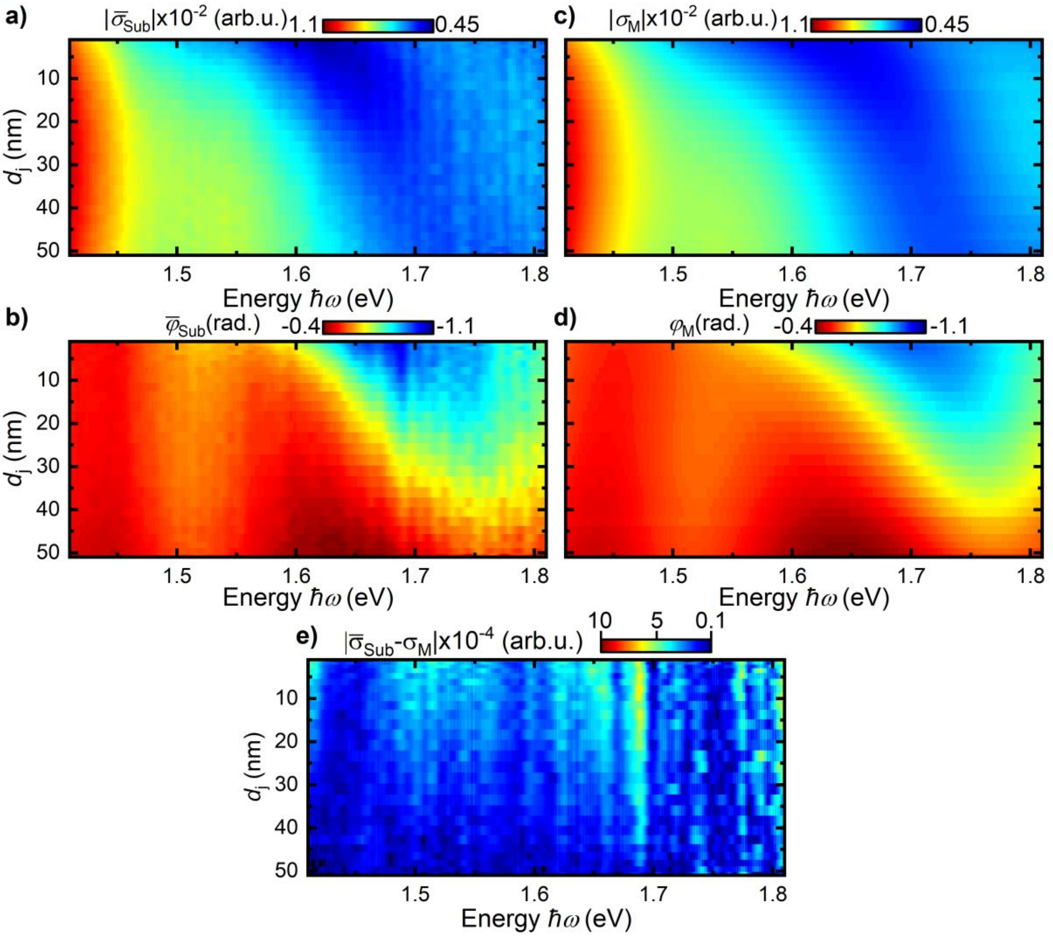

Figure S9: Response function $\overline{\sigma}(d_{\mathrm{j}},\hbar\omega)$ recorded when approaching the tip to a 90 nm SiO_2 layer on an Si substrate and fit to a three-oscillator model. $\overline{\sigma}$ is plotted as a function of the tip-sample distance d_{j} and the photon energy $\hbar\omega$. **a,b)** Amplitude $|\overline{\sigma}_{\mathrm{Sub}}|$ (**a**) and spectral phase $\overline{\varphi}_{\mathrm{Sub}}$ (**b**) of $\overline{\sigma}$. **c,d)** Amplitude $|\sigma_{\mathrm{M}}|$ (**c**) and spectral phase φ_{M} (**d**) obtained from a fit to the data. **e)** Deviation of the fit from the measured response function $|\overline{\sigma}_{\mathrm{Sub}} - \sigma_{\mathrm{M}}|$.

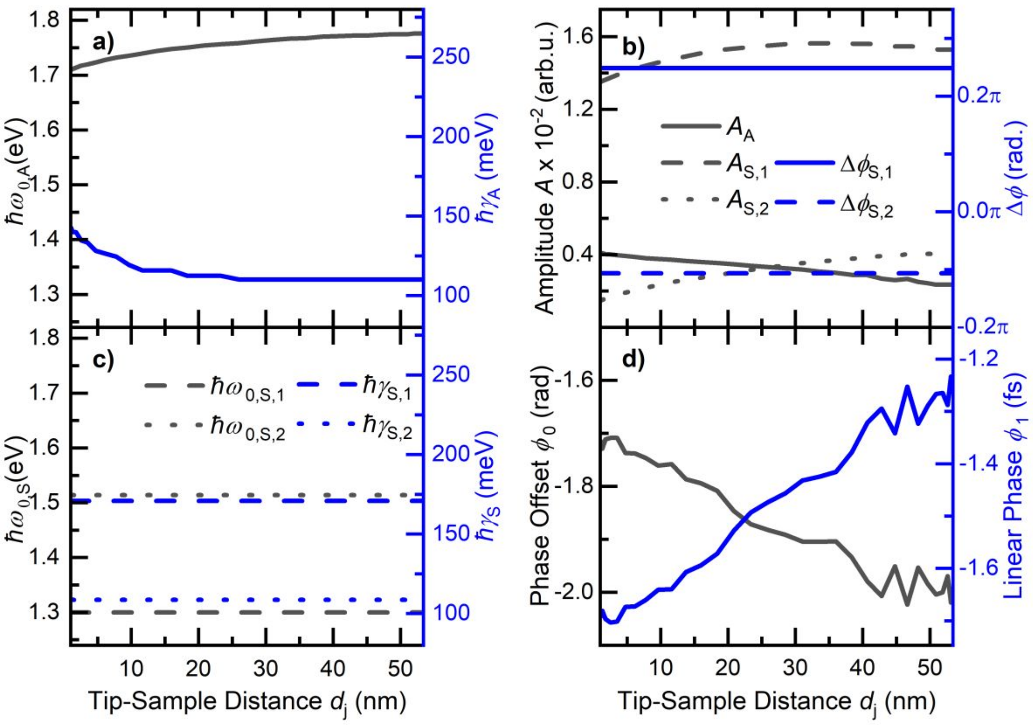

Figure S10: Resonance energy, linewidth and amplitude of the apex and shaft modes for the SiO_2/Si substrate as obtained from a fit to the data in Fig. S9. **a)** Distant-dependent resonance energy $\hbar\omega_{0,\mathrm{A}}$ (black line) and linewidth $\hbar\gamma_{\mathrm{A}}$ (blue line) of the apex mode. **b)** Amplitudes A_{A} (black solid line), $A_{\mathrm{S},1}$ (black dashed line) $A_{\mathrm{S},2}$ (black dotted line) of apex and shaft modes, respectively. The relative phase shifts $\Delta\phi_1$ and $\Delta\phi_2$ between oscillators are depicted as a solid and dashed blue line, respectively. **c)** Resonance energy $\hbar\omega_{0,\mathrm{S},1}$ (black dashed line) and linewidths $\hbar\gamma_{\mathrm{S},1}$ (blue dashed line), as well as resonance energy $\hbar\omega_{0,\mathrm{S},2}$ (black dotted line) and $\hbar\gamma_{\mathrm{S},2}$ (blue dotted line) of the shaft modes. **d)** Phase offset ϕ_0 (black line) and linear phase ϕ_1 (blue line).

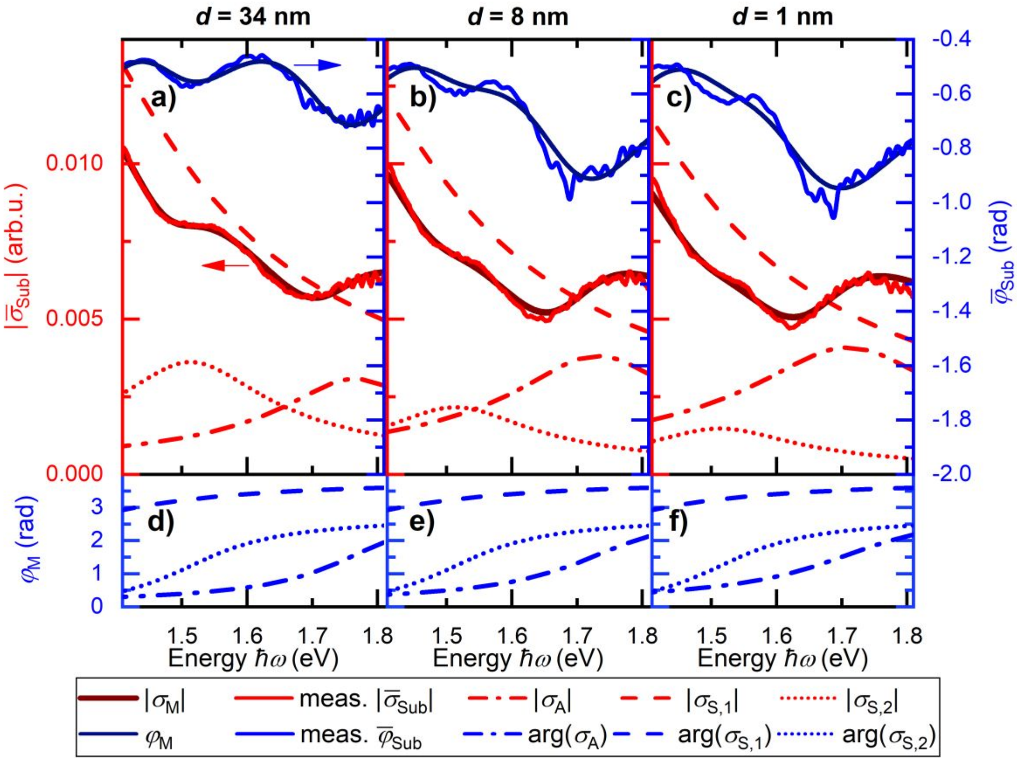

Figure S11: Response functions recorded on SiO_2/Si substrate at selected distance and fits to the Lorentz oscillator model. **a), b), c)** Measured amplitude $|\overline{\sigma}_{Sub}|$ (red) and spectral phase $\overline{\varphi}_{Sub}$ (blue) at distances d of 34, 8, and 1 nm, respectively. Fits are shown as dark red and dark blue lines. The amplitudes of the apex mode (dash-dotted lines) and the first (dashed lines) and second shaft mode (dotted lines) are added. **d), e), f)** Spectral phases φ_M of the three indivial modes as extracted from the model.

7. Point dipole model for the effective polarizability of the apex and shaft dipoles

Throughout the manuscript, we consider the electric fields emitted by the gold taper as those of a few virtual point-dipole emitters: a tip apex dipole and one or two shaft dipoles that are located on the taper axis at a finite distance from the apex. This simplified modelling of the tip emission is justified by electron energy loss[18, 19] and cathodoluminescence[20, 21] experiments that show that for such tips the emission from a dipolar mode located near the taper apex is spatially well separated from that of higher-order modes of the taper.[22]

Quite generally the dipole moment $\boldsymbol{p}_{\mathbf{d}}$ of a point-like emitter in the vicinity of a reflecting surface can be written as[2, 13, 23]

$$\boldsymbol{p}_{\mathbf{d}} = \overleftrightarrow{\boldsymbol{\alpha}}_{\mathbf{d}}\left(\boldsymbol{E}_{\mathbf{in}} + \frac{k_0^2}{\epsilon_0}\overleftrightarrow{\mathbf{G}}_{\mathbf{R}}\boldsymbol{p}_{\mathbf{d}}\right)(20)$$

Here, $\overleftrightarrow{\boldsymbol{\alpha}}_{\mathbf{d}}$ is the polarizability tensor of the emitter and $\boldsymbol{E}_{\mathbf{in}}$ is the incident field at the dipole position. The secondary field $\boldsymbol{E}_{\mathbf{R}} = \frac{k_0^2}{\epsilon_0}\overleftrightarrow{\mathbf{G}}_{\mathbf{R}}\boldsymbol{p}_{\mathbf{d}}$ is the field that is created by the dipole and reflected off the surface. The dyadic Greens function of the reflected field is $\overleftrightarrow{\mathbf{G}}_{\mathbf{R}}$ and k_0 denotes the magnitude of the wavevector of the incident field. By introducing an effective dipole polarizability tensor $\overleftrightarrow{\boldsymbol{\alpha}}_{\mathbf{eff}}$, Eq. (34) can be cast into a self-consistent expression for the dipole moment.

$$\boldsymbol{p}_{\mathbf{d}} = \frac{\overleftrightarrow{\boldsymbol{\alpha}}_{d}\boldsymbol{E}_{\mathbf{in}}}{\left(\overleftrightarrow{\mathbf{I}} - \frac{k_0^2}{\epsilon_0}\overleftrightarrow{\boldsymbol{\alpha}}_{\mathbf{d}}\overleftrightarrow{\mathbf{G}}_{\mathbf{R}}\right)} = \overleftrightarrow{\boldsymbol{\alpha}}_{\mathbf{eff}}\boldsymbol{E}_{\mathbf{in}}(21)$$

In free space, the electric field $\boldsymbol{E}(\boldsymbol{r})$ that is emitted from a point-dipole at position $\boldsymbol{r}_{\mathbf{0}}$ is

$$\boldsymbol{E}(\boldsymbol{r}) = \frac{k_0^2}{\epsilon_0}\overleftrightarrow{\mathbf{G}}_{\mathbf{0}}(\boldsymbol{r}_{\mathbf{0}},\boldsymbol{r})\boldsymbol{p}_{\mathbf{d}}(22)$$

Here $\overleftrightarrow{\mathbf{G}}_{\mathbf{0}}(\boldsymbol{r}_{\mathbf{0}},\boldsymbol{r}) = \overleftrightarrow{\mathbf{G}}_{\mathbf{NF}}(\boldsymbol{r}_{\mathbf{0}},\boldsymbol{r}) + \overleftrightarrow{\mathbf{G}}_{\mathbf{IF}}(\boldsymbol{r}_{\mathbf{0}},\boldsymbol{r}) + \overleftrightarrow{\mathbf{G}}_{\mathbf{FF}}(\boldsymbol{r}_{\mathbf{0}},\boldsymbol{r})$ denotes the free-space dyadic with near field, intermediate field and far field Green's functions

$$\overleftrightarrow{\mathbf{G}}_{\mathbf{NF}}(\boldsymbol{r}_{\mathbf{0}},\boldsymbol{r}) = \frac{\exp(ik_0R)}{4\pi R}\frac{1}{k_0^2R^2}\left[-\overleftrightarrow{\mathbf{I}} + \frac{3\boldsymbol{R}\boldsymbol{R}}{R^2}\right](23a)$$

$$\overleftrightarrow{\mathbf{G}}_{\mathbf{IF}}(\boldsymbol{r}_{\mathbf{0}},\boldsymbol{r}) = \frac{\exp(ik_0R)}{4\pi R}\frac{i}{k_0R}\left[\overleftrightarrow{\mathbf{I}} - \frac{3\boldsymbol{R}\boldsymbol{R}}{R^2}\right](23b)$$

$$\overleftrightarrow{\mathbf{G}}_{\mathbf{FF}}(\boldsymbol{r}_{\mathbf{0}},\boldsymbol{r}) = \frac{\exp(ik_0R)}{4\pi R}\left[\overleftrightarrow{\mathbf{I}} - \frac{\boldsymbol{R}\boldsymbol{R}}{R^2}\right](23c)$$

The vector from the dipole position to the observation point is $\boldsymbol{R} = \boldsymbol{r} - \boldsymbol{r_0}$ and its length is $R = |\boldsymbol{R}|$. $\overleftrightarrow{\mathbf{I}}$ denotes the identity matrix.

For the apex dipole, we assume that it is located in the center of a sphere with radius a_0 that mimics the shape of the taper apex. With d denoting the distance between apex and sample, with the interface placed in the plane $z = 0$, its position is $\boldsymbol{r_0} = (d + a_0)\hat{\mathbf{e}}_\mathbf{z}$. For a gold tapers fundamental mode, the dominating component of the polarizability tensor, α_{zz}, is oriented along the long axis of the taper, $\hat{\mathbf{e}}_\mathbf{z}$. We assume that only the component of the incident field pointing along $\hat{\mathbf{e}}_\mathbf{z}$ creates a dipole moment and that this dipole moment is also oriented along $\hat{\mathbf{e}}_\mathbf{z}$.[2] We thus take the polarizability of the apex dipole as $\overleftrightarrow{\boldsymbol{\alpha}}_\mathbf{A} = \alpha_{zz}\hat{\mathbf{e}}_\mathbf{z}\hat{\mathbf{e}}_\mathbf{z}$, with $\hat{\mathbf{e}}_\mathbf{z}\hat{\mathbf{e}}_\mathbf{z}$ being the outer product. Using the Weyl identity to decompose the tip field into plane waves with in-plane moment $k_\parallel$, the apex dipole moment can then be written as [6, 13, 24]:

$$p_{A,z}\hat{\mathbf{e}}_\mathbf{z} = \frac{\alpha_{zz}}{\left(1 - \frac{\alpha_{zz}}{4\pi\epsilon_0}\int_0^\infty k_\parallel^2 e^{-2k_\parallel(d+a_0)} r^p\left(k_\parallel\right) dk_\parallel\right)} E_{in,z}\,\hat{\mathbf{e}}_\mathbf{z} (24)$$

Here $r_{TM}\left(k_\parallel\right)$ is the Fresnel reflection coefficient of the surface for p-polarized light. Explicit calculation of the Fresnel coefficients for the gold and dielectric substrates studied in this work in Section 9 shows that is justified to replace Eq. 24 by its quasistatic limit, i.e., to consider only near-field reflection in the limit $k_\parallel \rightarrow \infty$. This gives the reflection coefficient $\beta = r_{TM}\left(k_\parallel \rightarrow \infty\right) = (\epsilon - 1)/(\epsilon + 1)$ where ϵ is the dielectric function of the top-most surface layer.

This results in

$$\boldsymbol{p}_\mathbf{d} = p_{A,z}\hat{\mathbf{e}}_\mathbf{z} = \alpha_{eff} E_{in,z}\,\hat{\mathbf{e}}_\mathbf{z} = \frac{\alpha_{zz}}{1 - \frac{\beta\alpha_{zz}}{16\pi\epsilon_0(d + a_0)^3}} E_{in,z}\,\hat{\mathbf{e}}_\mathbf{z} (25)$$

with α_{eff} being the effective polarizability of the tip sample junction. For a conical gold taper as it is used in experiment, it is not sufficient to estimate the polarizability α_{zz} of the gold taper by the polarizability of a small sphere with radius a_0 [25, 26] since localized surface plasmon excitation in the vicinity of the taper can couple to surface plasmon polariton (SPP) modes that propagate along the taper shaft.[18, 19, 27, 28]. We therefore phenomenologically approximate the polarizability of the taper by a Lorentzian line shape[2, 25] $\alpha_{\text{zz}}(\omega) = \alpha L_{\text{A}}(\omega,\omega_0,\gamma)$, characterized by a resonance frequency ω_0, linewidth γ and a scaling parameter α. The scaling parameter allows us to adjust the local near-field enhancement $F(\omega,d) = E_{\text{A}}(\boldsymbol{r}_{\mathbf{A}},\omega)/E_{\text{in,z}}(\boldsymbol{r}_{\mathbf{0}},\omega)$ of the taper, i.e., the ratio of the field amplitude $\boldsymbol{E}_{\mathbf{A}}\left(\boldsymbol{r}_{\mathbf{A}} = d\hat{\mathbf{e}}_{\mathbf{z}}\right)$ at the very apex of the tip and the z-component of the incident field $E_{\text{in,z}}(\boldsymbol{r}_{\mathbf{0}})$. For large tip-sample distances $d \gg a_0$ the amplitude of the local field enhancement

$$|F(\omega)| = \frac{|\alpha_{\text{zz}}(\omega)|}{2\pi\epsilon_0 a_0^3}(26)$$

only depends on the polarizability α_{zz} and tip radius a_0. For gold tapers with radii a_0 between 8 and 12 nm and large half opening angles ($\theta \approx 16°$) the field enhancement in the wavelength range of 800 nm is typically $|F| \approx 10 - 20$.[29] For such large tip-sample distances, i.e., negligible tip-sample coupling, the maximum field enhancement $|FE| = |F(\text{d}\rightarrow\infty,\omega_0)|$ is reached at ω_0, and FE can be considered as a material property of the gold taper. For shorter distances, near-field coupling affects the field enhancement, making its maximum value $F_{\text{m}}(d) = \max_{\omega} F(d,\omega)$ dependent on the tip-sample distance d.

Numerical simulations based on the Multiple Multipole Method[13] and on Finite-Difference Time-Domain (FDTD) calculations[29] indicate that is not sufficient to consider F as a real-

valued number but that it is important to allow for a finite phase shift between incident field and field at the apex. This is physically intuitive since the localized apex field is coupled to propagating SPP modes of the shaft, effectively introducing a Fano-like phase shift of the apex mode.[30] We incorporate such a phase shift in our model by allowing for a complex-valued field enhancement factor

$$FE = |FE| e^{i\phi_{\mathrm{FE}}} (27)$$

With this, $\alpha = 2\pi\epsilon_0 a_0^3\, FE$ and

$\alpha_{\mathrm{zz}}(\omega) = 2\pi\epsilon_0 a_0^3\, FE\, L_{\mathrm{A}}(\omega,\omega_0,\gamma) (28)$

This describes the frequency dependence of the tip polarizability and, using Eq. (25) gives the effective frequency-dependent polarizability of the tip-sample junction as a function of d. Eq. (28) is used to evaluate the frequency-dependent tip polarizability from the distant-dependent response functions shown in Section 6.

For an intuitive understanding of the tip-sample coupling, it is instructive to introduce $q(\omega,d) = \frac{\alpha_{\mathrm{zz}}(\omega)\beta(\omega)}{16\pi\epsilon_0(d+a_0)^3}$ and to express the effective polarizability in terms of a geometric series

$$\alpha_{\mathrm{eff}}(\omega,d) = \alpha_{\mathrm{zz}}\left(\frac{1}{1-q}\right) = \alpha_{\mathrm{zz}}\sum_{n=0}^{\infty} q^n \ (29)$$

This gives the effectively polarizability in a perturbative expansion of multiple near-field reflections between tip and sample. We found this helpful in understanding the effect of ϕ_{FE} on the lineshape of the electric field that is emitted by the apex mode. For sufficiently large distances d, the induced dipole moment is proportional to the 0th order polarizability $\alpha^{(0)} = \alpha_{\mathrm{zz}}$. In our experiments, this is typically observed for $d >$ 30 nm. When the gold tip approaches the sample surface, higher orders of the polarizability $\alpha^{(n)} = \alpha_{\mathrm{zz}} q^n$ need to be considered in

the effective polarizability. For distances d that fulfill $q(\omega,d) < 1$, $\alpha^{(n)} < \alpha^{(n-1)}$ a perturbative expansion is justified. In this case, the effect of ϕ_{FE} and the tip-sample coupling on the lineshape of the apex mode can be understood by considering the 1st order perturbation. Figure S12 compares the spectral phase of $\arg(\alpha^{(0)})$ and $\arg(\alpha^{(1)})$ for a real valued field enhancement ($\phi_{\mathrm{FE}} = 0\pi$) and for a phase shift of $\phi_{\mathrm{FE}} = 0.2\pi$. The spectral shape of α_{eff} depends on the interference between both contributions and thus on the phase difference $\arg(\alpha^{(1)}) - \arg(\alpha^{(0)})$ (Fig. S12e,f). The condition for constructive interference is depicted by a shaded red background while destructive interference is marked in shaded blue. In the absence of a phase shift ϕ_{FE}, constructive interference appears for $\omega < \omega_0$ while the two polarizabilities interfere destructively for $\omega > \omega_0$. This induces a net red shift of the apex resonance without affecting its linewidth. In contrast, for $\phi_{\mathrm{FE}} = 0.5\pi$, destructive interference is seen for all frequencies. Hence, the resonance frequency remains effectively unchanged while the linewidth of the apex resonance increases due to the tip-sample coupling. For intermediate cases, $0 < \phi_{\mathrm{FE}} < 0.5\pi$, the tip-sample coupling leads to both spectral redshifts and line broadenings. This results in a decrease in the maximum field enhancement upon approach to the sample. For small tip-sample distances, a perturbation expansion is no longer justified[2] and the self-consistent solution of Eq. (29) needs to be considered. In our experiments, this is typically the case for tip-sample distances of a few nanometer.

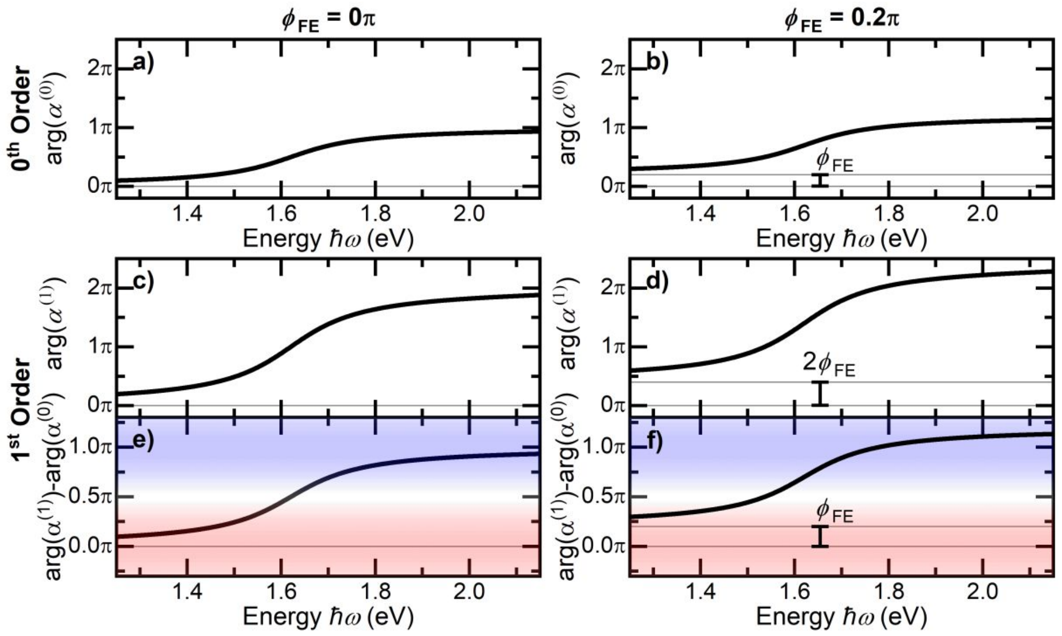

Figure S12: Perturbative expansion of the tip-sample coupling. **a,b)** Spectral phase of the 0th-order polarizability $\arg(\alpha^{(0)})$ for $\phi_{\mathrm{FE}} = 0$ (**a**) and 0.2π (**b**), respectively. **c,d)** Spectral phase of the 1th-order polarizability $\arg(\alpha^{(1)})$ for $\phi_{\mathrm{FE}} = 0$ (**c**) and 0.2π (**d**), **e,f)** Phase difference $\arg(\alpha^{(1)}) - \arg(\alpha^{(0)})$ for $\phi_{\mathrm{FE}} = 0$ (**e**) and 0.2π (**f**), respectively. For $\phi_{\mathrm{FE}} = 0$, constructive interference (shaded red) is seen for $\omega < \omega_0$ while destructive interference (shaded blue) appears for higher frequencies. Thus, $\alpha^{(1)}$ results in a red shift of the tip resonance. For $\phi_{\mathrm{FE}} = 0.2\,\pi$, destructive interference induces a line broadening.

The same approach can also be used to describe the shaft dipoles. Since these correspond to emission from higher order modes of the taper, specifically from the shaft region in which they are converted from a bound to an unbound mode,[18, 22, 31] we assume that their effective dipole moments are oriented along the sample surface. Since the associated electric fields are emitted from regions that are separated from the apex by a few 100 nm,[18, 20] emitter-surface coupling mostly results from reflections of propagating fields at the sample surface while near-field coupling is negligible. The effect of the reflected light on the resonance energy and linewidth of the shaft modes is weak and can be neglected. Mostly, destructive interference between the

field emitted by the shaft dipole and the reflected field results in destructive interference when approaching the surface.

8. Momentum dependent generalized Fresnel coefficients

To justify the quasi-static approximation for the apex polarizability, Fresnel reflection coefficients for p-polarized, transverse magnetic light r_{TM} are calculated for a planar air-gold interface and for the SiO_2 on Si multilayer structure investigated experimentally (Figs. S13 and S14). The reflection coefficients are obtained as a function of in-plane momentum $k_\parallel$[23] and photon energy $\hbar\omega$ using published dielectric functions for gold,[32] silicon[33] and silicon dioxide.[34] For the air-gold interface bulk Fresnel coefficients are calculated and for the air-SiO_2-Si layer structure a transfer matrix method is used to calculate the generalized momentum dependent Fresnel reflection coefficients.[23] The thickness of the SiO_2 layer is 90 nm. Momentum- and photon-energy resolved map of r_{TM} for the gold (Fig. S13a) and the dielectric layer structure (Fig. S14a). The quasistatic reflection coefficients $\beta = r_{\mathrm{TM}}(k_\parallel \to \infty)$ are depicted in Figs. S13b and S14b for the two interfaces. An important part of the integrand in Eq. (24) is the coupling weight function (CWF)

$$CWF = k_\parallel^2 e^{-2k_\parallel(d+a_o)} \quad (48)$$

The CWF is depicted in Figs. S13c and S14c for the two interfaces for different tip-sample distances d. The scaling of the CWF with $k_\parallel^2$ effectively insures that the integral in Eq. (24) can be replaced by its quasi-static limit.

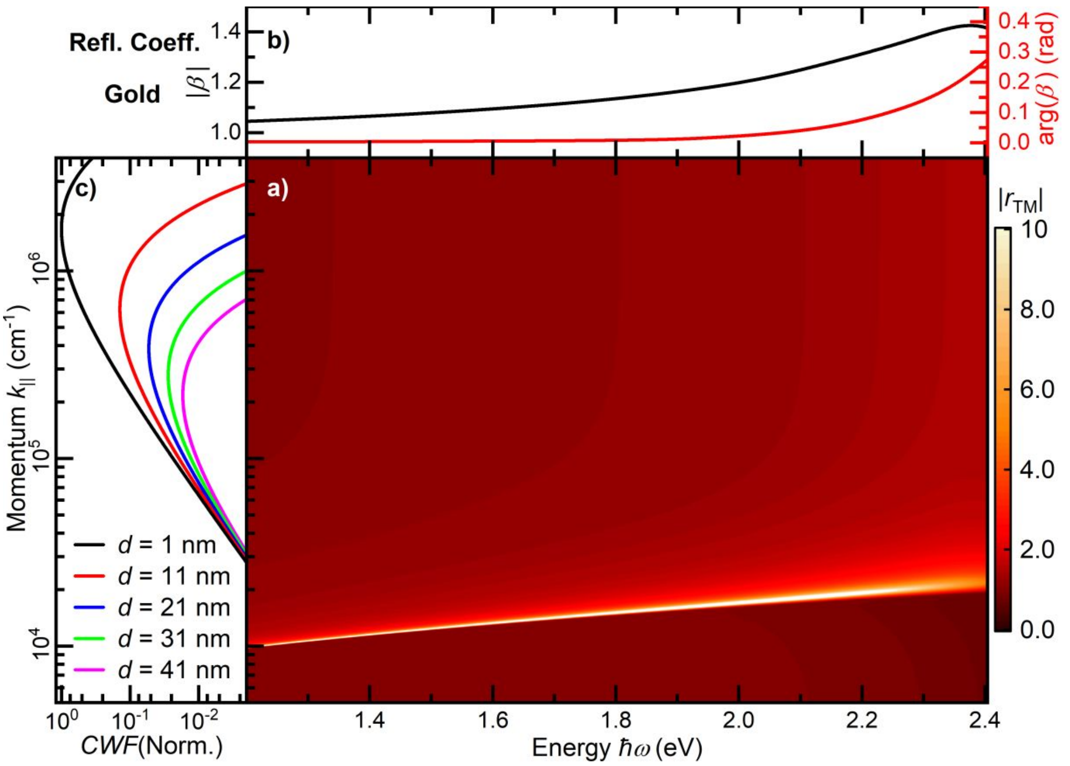

Figure S13: Reflection coefficients of a planar gold/air interface for propagating and evanescent fields. **a)** Magnitude of the reflection coefficient $|r_{\mathrm{TM}}|$ for p-polarized, transverse magnetic light as a function of in-plane momentum $k_{\|}$ and photon energy. For $k_{\|} \cong 10^4$ cm^{-1}, the light line and the surface plasmon peak are seen. For larger momenta, the reflection coefficient converges against the quasi-static reflection coefficient $|\beta|$. **b)** Quasi-static reflection coefficient $|\beta|$ as a function of photon energy. **c)** Coupling weight functions $CWF(k_{\|}$ $,d)$ for different tip-sample distances d.

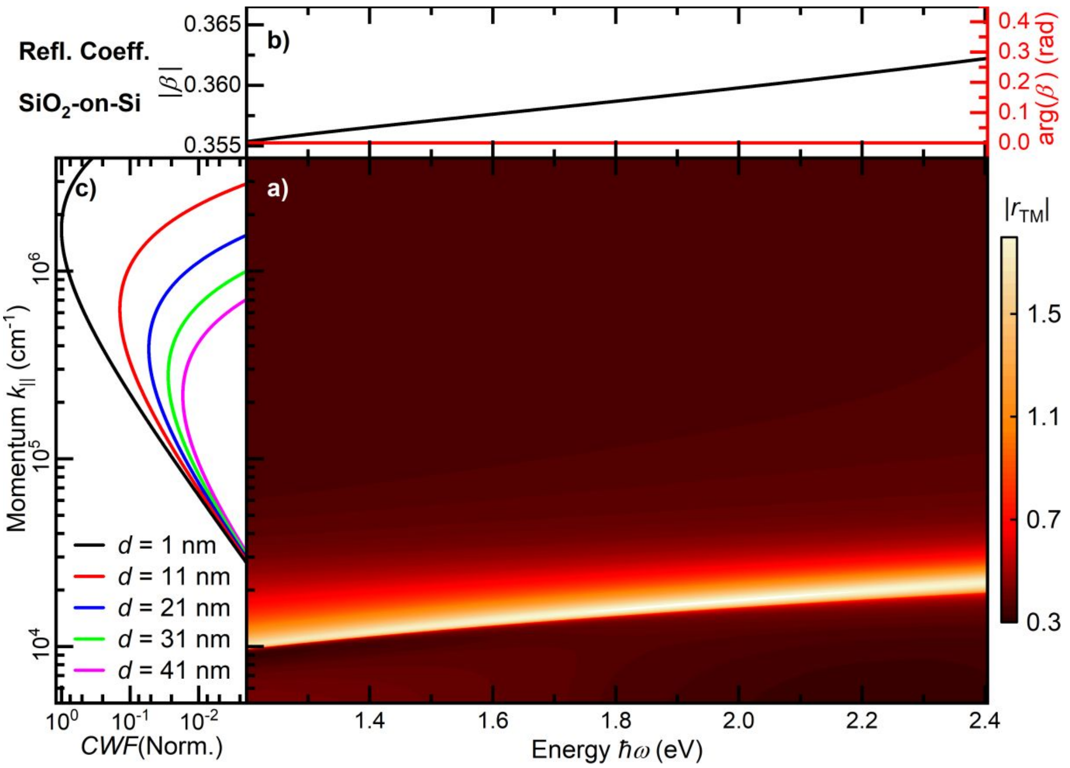

Figure S14: Reflection coefficient of the interface between air and a 90-nm thick SiO_2 layer on a Si substrate for propagating and evanescent fields. **a)** Magnitude of the reflection coefficient $|r_{\mathrm{TM}}|$ for p-polarized, transverse magnetic light as a function of in-plane momentum $k_{\|}$ and photon energy. For larger momenta, the reflection coefficient converges against $|\beta|$. **b)** Quasi-static reflection coefficient $|\beta|$ as a function of photon energy. **c)** Coupling weight functions $CWF(k_{\|},d)$ for different tip-sample distances d.

9. Sensitivity of the effective polarizability model

We use the Lorentz oscillator model, Eqs. (18) and (19), to evaluate how amplitude, resonance energy and linewidth of the apex oscillator change upon approaching the gold surface. The results of this analysis are shown in Fig. S8. This allows us to estimate $\omega_{0,\mathrm{A}}$ $(d\rightarrow\infty)$, and $\gamma_{\mathrm{A}}(d\rightarrow\infty)$ in the limit of large tip-sample distances. Using these parameters, we can now take Eq. 29 to predict, for a given set of field enhancement FE and apex radius a_0, how $\alpha_{\mathrm{eff}}(\omega,d)$ changes with distance d. From this two-dimensional data, we extract distance-dependent curves of the predicted amplitudes, resonance energies and linewidths. We compare

the extracted parameters (Fig. S8) and the values predicted by Eq. 29 for each of the three quantities. From this comparison we deduce a coefficient of determination R^2 for each parameter set (FE, a_0). We find an optimum set of parameters ($\phi_{\mathrm{FE}} = 0.18\pi$, $a_0 =$ 10 nm, and $|FE| = 17.8$, giving an R^2 value of 0.93. We now want to estimate the sensitivity of R^2 to the most relevant free parameters of the model, i.e., the magnitude $|FE|$, the phase ϕ_{FE} and the apex radius a_0. For this, we show, in Fig. S15, how R^2 depends on two of these parameters while keeping the third one fixed at its optimum value. From these data we estimate that we can deduce each of the three parameters with an accuracy of approximately $\pm 10\%$.

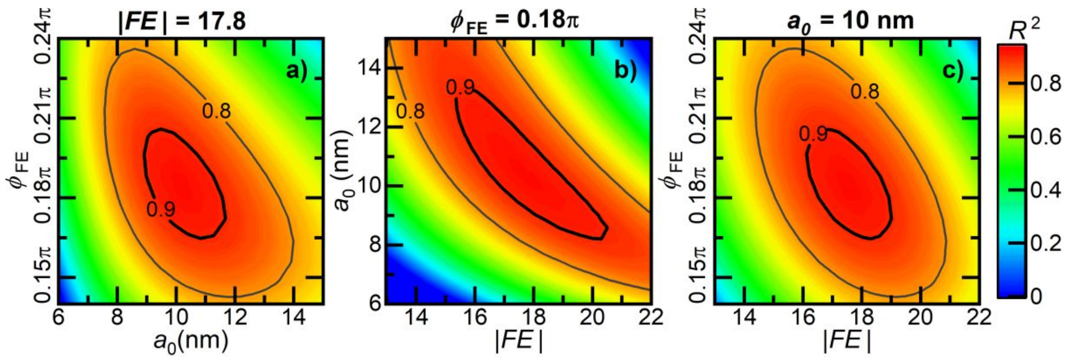

Figure S15: Sensitivity of the effective apex polarizability $\alpha_{\mathrm{eff}}(FE,a_0)$ on the parameters FEH and a_0. To deduce this sensitivity, we compare the distance-dependent parameters $\omega_{0,\mathrm{A}}$,γ_{A} and A_{A} from Fig. S8 with the values predicted by Eq. (29) for a given set of parameters FE and a_0. In the three panels, it is shown how the coefficient of determination R^2 that results from this comparison depends on two of these parameters while keeping the third one fixed at its optimum value. **a)** $R^2(a_0, \phi_{\mathrm{FE}})$ for $|FE| = 17.8$; **b)** $R^2(|FE|,a_0)$ for $\phi_{\mathrm{FE}} = 0.18\pi$; **c)** $R^2(|FE|, \phi_{\mathrm{FE}})$ for $a_0 = 10$ nm.

References

(1) Zhan, J.; Jehle, T.; Stephan, S.; Tiguntseva, E.; Nochowitz, S. S.; Groß, P.; Duan, J.; Makarov, S.; Lienau, C. Interferometric Near-field Fano Spectroscopy of Single Halide Perovskite Nanoparticles. *Nano Lett.* **2024**, *24* (49), 15738-15744.
(2) Esmann, M.; Becker, S. F.; Witt, J.; Zhan, J.; Chimeh, A.; Korte, A.; Zhong, J.; Vogelgesang, R.; Wittstock, G.; Lienau, C. Vectorial near-field coupling. *Nat. Nanotechnol.* **2019**, *14* (7), 698-704.

(3) Esmann, M.; Chimeh, A.; Korte, A.; Zhong, J.-H.; Stephan, S.; Witt, J.; Wittstock, G.; Talebi, N.; Lienau, C. Plasmonic nanofocusing spectral interferometry. *Nanophotonics* **2020**, *9* (2), 491-508.
(4) Zhong, J. H.; Chimeh, A.; Korte, A.; Schwarz, F.; Yi, J. M.; Wang, D.; Zhan, J. X.; Schaaf, P.; Runge, E.; Lienau, C. Strong Spatial and Spectral Localization of Surface Plasmons in Individual Randomly Disordered Gold Nanosponges. *Nano Lett.* **2018**, *18* (8), 4957-4964.
(5) Schmidt, S.; Piglosiewicz, B.; Sadiq, D.; Shirdel, J.; Lee, J. S.; Vasa, P.; Park, N.; Kim, D.-S.; Lienau, C. Adiabatic Nanofocusing on Ultrasmooth Single-Crystalline Gold Tapers Creates a 10-nm-Sized Light Source with Few-Cycle Time Resolution. *Acs Nano* **2012**, *6* (7), 6040-6048.
(6) Becker, S. F.; Esmann, M.; Yoo, K.; Gross, P.; Vogelgesang, R.; Park, N.; Lienau, C. Gap-Plasmon-Enhanced Nanofocusing Near-Field Microscopy. *ACS Photonics* **2016**, *3* (2), 223-232.
(7) Stibenz, G.; Ropers, C.; Lienau, C.; Warmuth, C.; Wyatt, A. S.; Walmsley, I. A.; Steinmeyer, G. Advanced methods for the characterization of few-cycle light pulses: a comparison. *Appl. Phys. B* **2006**, *83* (4), 511-519.
(8) Stibenz, G.; Steinmeyer, G. Interferometric frequency-resolved optical gating. *Opt. Express* **2005**, *13* (7), 2617-2626.
(9) Zhong, J.-H.; Vogelsang, J.; Yi, J.-M.; Wang, D.; Wittenbecher, L.; Mikaelsson, S.; Korte, A.; Chimeh, A.; Arnold, C. L.; Schaaf, P.; et al. Nonlinear plasmon-exciton coupling enhances sum-frequency generation from a hybrid metal/semiconductor nanostructure. *Nat. Commun.* **2020**, *11* (1), 1464.
(10) Knoll, B.; Keilmann, F. Enhanced dielectric contrast in scattering-type scanning near-field optical microscopy. *Opt. Commun.* **2000**, *182* (4), 321-328.
(11) Hillenbrand, R.; Keilmann, F. Complex Optical Constants on a Subwavelength Scale. *Phys. Rev. Lett.* **2000**, *85* (14), 3029-3032.
(12) Raschke, M. B.; Lienau, C. Apertureless near-field optical microscopy: Tip-sample coupling in elastic light scattering. *Appl. Phys. Lett.* **2003**, *83* (24), 5089-5091.
(13) Novotny, L.; Hecht, B. *Principles of Nano-Optics*; Cambridge University Press, 2012. DOI: DOI: 10.1017/CBO9780511794193.
(14) Lepetit, L.; Chériaux, G.; Joffre, M. Linear techniques of phase measurement by femtosecond spectral interferometry for applications in spectroscopy. *J. Opt. Soc. Am. B* **1995**, *12* (12), 2467-2474.
(15) Reynaud, F.; Salin, F.; Barthelemy, A. Measurement of phase shifts introduced by nonlinear optical phenomena on subpicosecond pulses. *Opt. Lett.* **1989**, *14* (5), 275-277.
(16) Lepetit, L.; Cheriaux, G.; Joffre, M. Linear techniques of phase measurement by femtosecond spectral interferometry for applications in spectroscopy. *J. Opt. Soc. Am. B* **1995**, *12* (12), 2467-2474.
(17) William, H. P.; Teukolsky, S. A.; Vetterling, W. T.; Flannery, B. P. *Numerical recipes 3rd edition: The art of scientific computing*; Cambridge University Press, 2007.
(18) Talebi, N.; Sigle, W.; Vogelgesang, R.; Esmann, M.; Becker, S. F.; Lienau, C.; van Aken, P. A. Excitation of Mesoscopic Plasmonic Tapers by Relativistic Electrons: Phase Matching versus Eigenmode Resonances. *Acs Nano* **2015**, *9* (7), 7641-7648.
(19) Schröder, B.; Weber, T.; Yalunin, S. V.; Kiel, T.; Matyssek, C.; Sivis, M.; Schäfer, S.; von Cube, F.; Irsen, S.; Busch, K.; et al. Real-space imaging of nanotip plasmons using electron energy loss spectroscopy. *Phys. Rev. B* **2015**, *92* (8), 085411.
(20) Guo, S.; Talebi, N.; Sigle, W.; Vogelgesang, R.; Richter, G.; Esmann, M.; Becker, S. F.; Lienau, C.; van Aken, P. A. Reflection and Phase Matching in Plasmonic Gold Tapers. *Nano Lett.* **2016**, *16* (10), 6137-6144.
(21) Guo, S.; Talebi, N.; Campos, A.; Sigle, W.; Esmann, M.; Becker, S. F.; Lienau, C.; Kociak, M.; van Aken, P. A. Far-Field Radiation of Three-Dimensional Plasmonic Gold Tapers near Apexes. *ACS Photonics* **2019**, *6* (10), 2509-2516.

(22) Esmann, M.; Becker, S. F.; da Cunha, B. B.; Brauer, J. H.; Vogelgesang, R.; Gross, P.; Lienau, C. k-space imaging of the eigenmodes of sharp gold tapers for scanning near-field optical microscopy. *Beilstein J. Nanotechnol.* **2013**, *4*, 603-610.
(23) Hohenester, U. *Nano and quantum optics*; Springer Nature, 2019. DOI: 10.1007/978-3-030-30504-8.
(24) McLeod, A. S.; Kelly, P.; Goldflam, M. D.; Gainsforth, Z.; Westphal, A. J.; Dominguez, G.; Thiemens, M. H.; Fogler, M. M.; Basov, D. N. Model for quantitative tip-enhanced spectroscopy and the extraction of nanoscale-resolved optical constants. *Phys. Rev. B* **2014**, *90* (8), 085136.
(25) Aizpurua, J.; Apell, S. P.; Berndt, R. Role of tip shape in light emission from the scanning tunneling microscope. *Phys. Rev. B* **2000**, *62* (3), 2065-2073.
(26) Behr, N.; Raschke, M. B. Optical Antenna Properties of Scanning Probe Tips: Plasmonic Light Scattering, Tip−Sample Coupling, and Near-Field Enhancement. *J. Phys. Chem. C* **2008**, *112* (10), 3766-3773.
(27) Ropers, C.; Neacsu, C. C.; Elsaesser, T.; Albrecht, M.; Raschke, M. B.; Lienau, C. Grating-Coupling of Surface Plasmons onto Metallic Tips: A Nanoconfined Light Source. *Nano Lett.* **2007**, *7* (9), 2784-2788.
(28) Neacsu, C. C.; Berweger, S.; Olmon, R. L.; Saraf, L. V.; Ropers, C.; Raschke, M. B. Near-Field Localization in Plasmonic Superfocusing: A Nanoemitter on a Tip. *Nano Lett.* **2010**, *10* (2), 592-596.
(29) Thomas, S.; Wachter, G.; Lemell, C.; Burgdörfer, J.; Hommelhoff, P. Large optical field enhancement for nanotips with large opening angles. *New J. Phys.* **2015**, *17* (6), 063010.
(30) Genet, C.; van Exter, M. P.; Woerdman, J. P. Fano-type interpretation of red shifts and red tails in hole array transmission spectra. *Opt. Commun.* **2003**, *225* (4), 331-336.
(31) Lee, J. S.; Han, S.; Shirdel, J.; Koo, S.; Sadiq, D.; Lienau, C.; Park, N. Superfocusing of electric or magnetic fields using conical metal tips: effect of mode symmetry on the plasmon excitation method. *Opt. Express* **2011**, *19* (13), 12342-12347.
(32) Johnson, P. B.; Christy, R. W. Optical Constants of the Noble Metals. *Phys. Rev. B* **1972**, *6* (12), 4370-4379.
(33) Malitson, I. H. Interspecimen Comparison of the Refractive Index of Fused Silica*,†. *J. Opt. Soc. Am.* **1965**, *55* (10), 1205-1209.
(34) Schinke, C.; Christian Peest, P.; Schmidt, J.; Brendel, R.; Bothe, K.; Vogt, M. R.; Kröger, I.; Winter, S.; Schirmacher, A.; Lim, S.; et al. Uncertainty analysis for the coefficient of band-to-band absorption of crystalline silicon. *AIP Adv.* **2015**, *5* (6), 067168.
